# Supplementary material for: Reversine, a selective MPS1 inhibitor, induced autophagic cell death via diminished glucose uptake and ATP production in cholangiocarcinoma cells
Source: PeerJ. 2021 Jan 7;9:e10637. doi: 10.7717/peerj.10637 (PMC7797171; doi:10.7717/peerj.10637)
Supplement: Figure S1 — CCA cells were treated with 0.25, 2.5, 25 and 250 µM of 5-FU for 48 h. Cells treated with 0.1% DMSO were used as a vehicle control. The cell viability was determined using the MTT assay. Data are mean ± SD of three independent experiments. [file peerj-09-10637-s001.docx]

**Supplementary Fig S1. Dose response curve of 5-FU in CCA cell lines**

CCA cells were treated with 0.25, 2.5, 25 and 250 µM of 5-FU for 48 h. Cells treated with 0.1% DMSO were used as a vehicle control. The cell viability was determined using the MTT assay. Data are mean ± SD of three independent experiments.
