# Supplementary material for: Reversine, a selective MPS1 inhibitor, induced autophagic cell death via diminished glucose uptake and ATP production in cholangiocarcinoma cells
Source: PeerJ. 2021 Jan 7;9:e10637. doi: 10.7717/peerj.10637 (PMC7797171; doi:10.7717/peerj.10637)
Supplement: Table S1 — IC50: Half-maximal inhibitory concentration. * P-value < 0.05 compared with reversine. [file peerj-09-10637-s002.docx]

**Supplementary Table S1.** IC_50_ of 5-FU in CCA cell lines

|  | **IC_50_ (µM) at 48 h** | |
| --- | --- | --- |
| **Cell line** | **Reversine** | **5-FU** |
| KKU-100 | 2.72±0.32 | >250 |
| KKU-213A | 6.83±2.61 | 41.59 ± 15.79***** |
| KKU-213B | 4.67±0.77 | 59.73 ± 35.12***** |

IC_50_: Half-maximal inhibitory concentration. * P-value <0.05 compared with reversine.
