# Supplementary material for: Reversine, a selective MPS1 inhibitor, induced autophagic cell death via diminished glucose uptake and ATP production in cholangiocarcinoma cells
Source: PeerJ. 2021 Jan 7;9:e10637. doi: 10.7717/peerj.10637 (PMC7797171; doi:10.7717/peerj.10637)
Supplement: Supplemental Information 6 [file peerj-09-10637-s006.zip › Original figure 2B and 3B.pptx]

## Slide 1
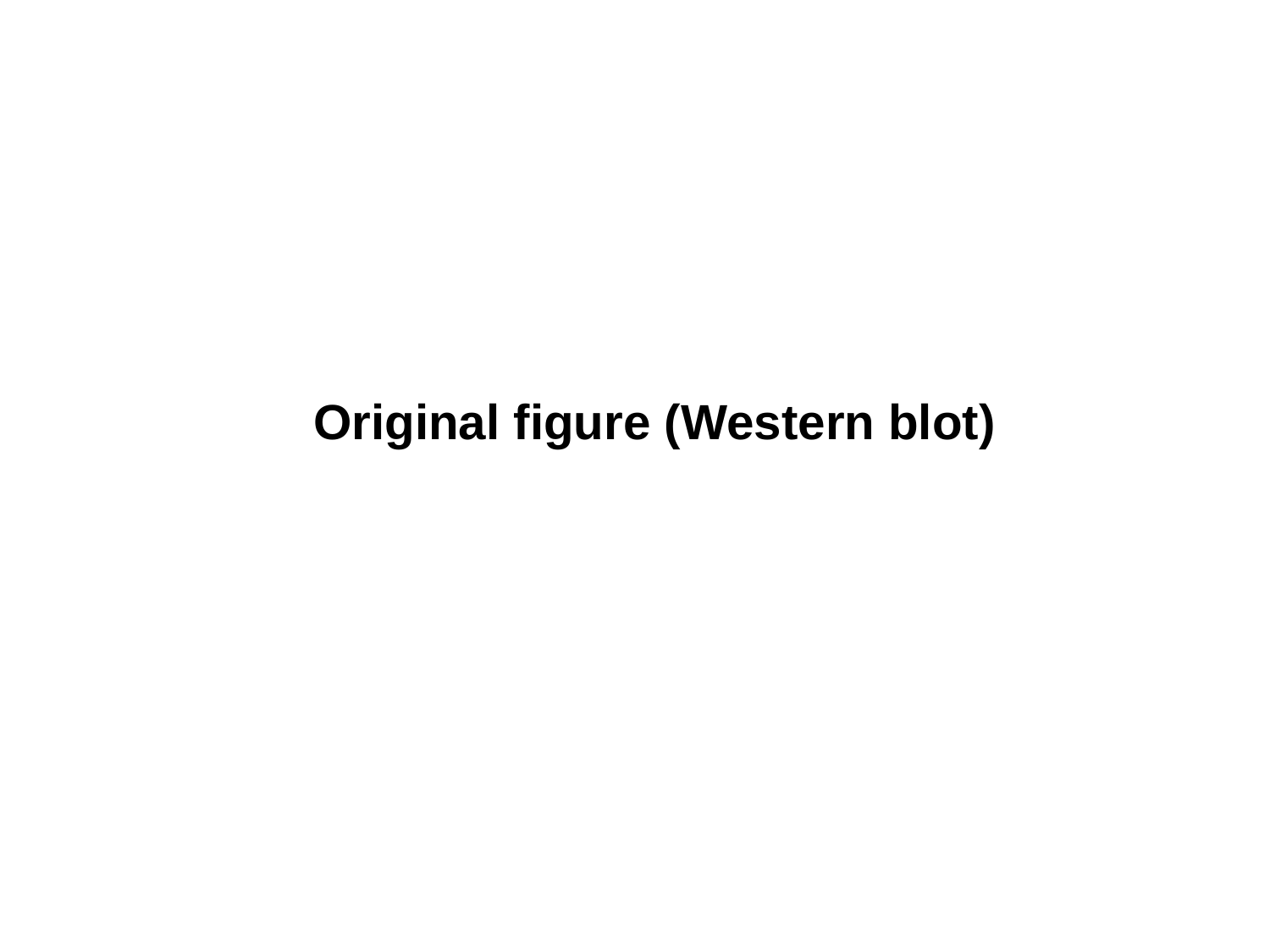

Original figure (Western blot)

## Slide 2
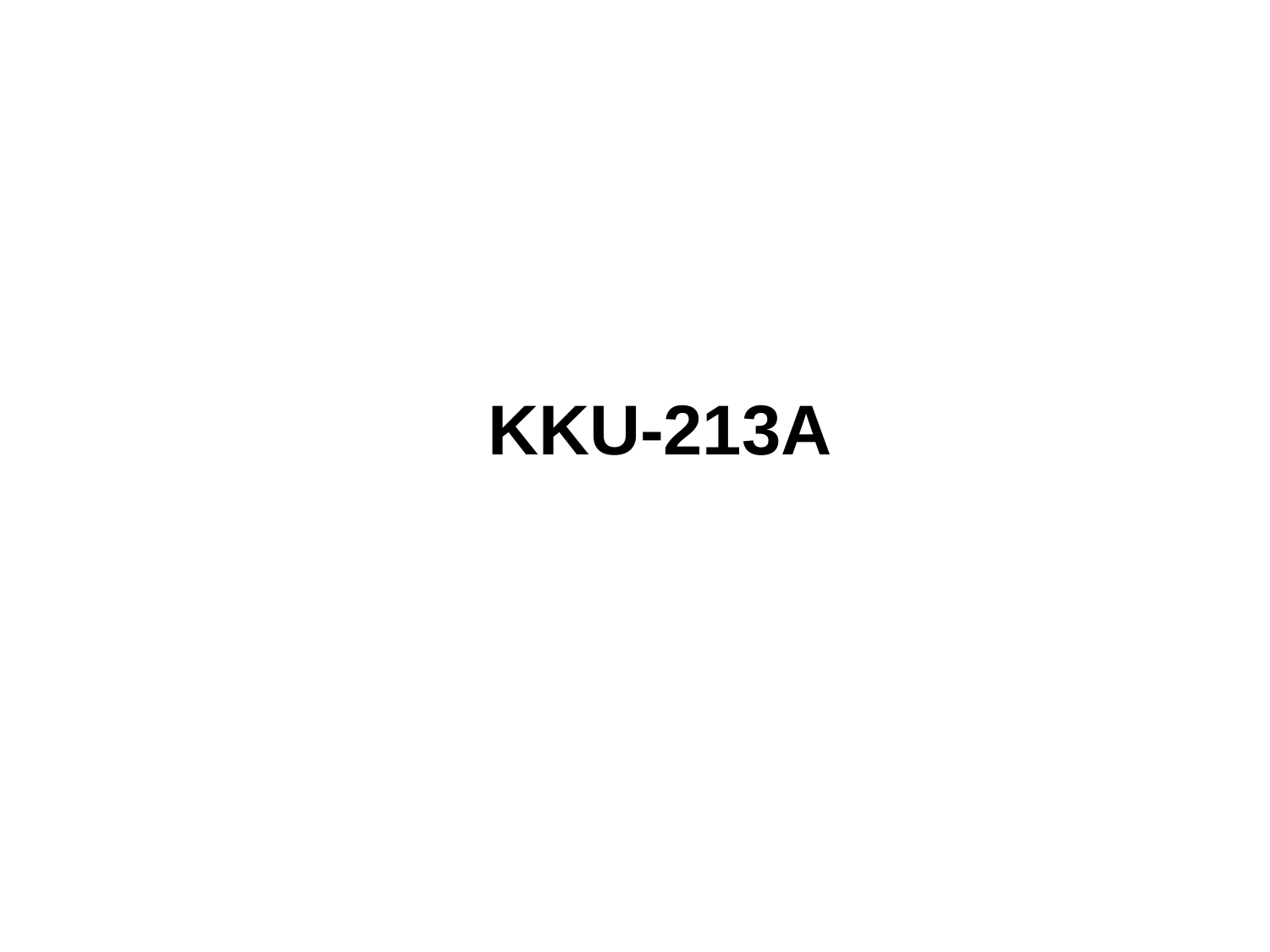

KKU-213A

## Slide 3
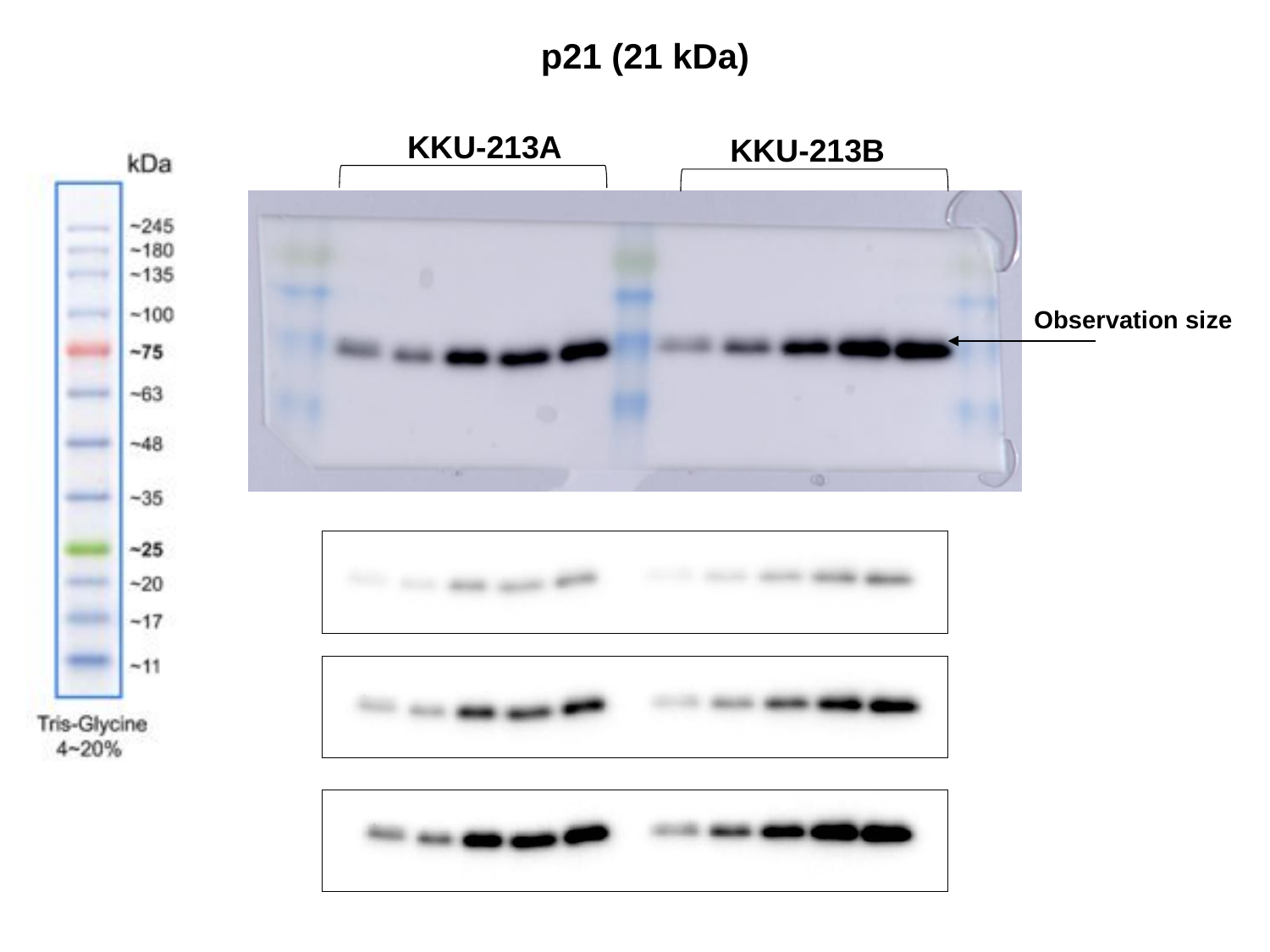

p21 (21 kDa)
KKU-213A
KKU-213B
Observation size

## Slide 4
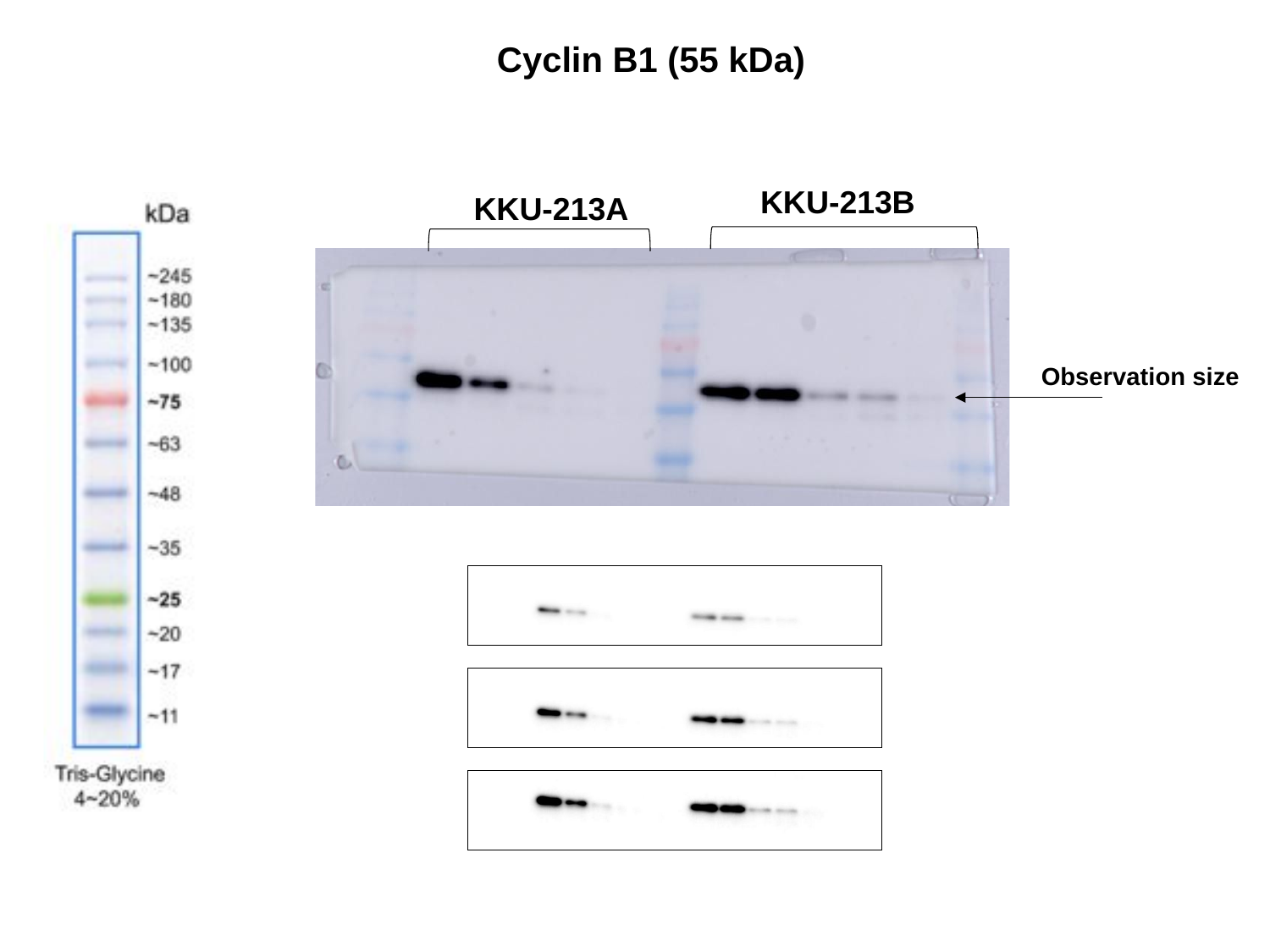

Cyclin B1 (55 kDa)
KKU-213B
KKU-213A
Observation size

## Slide 5
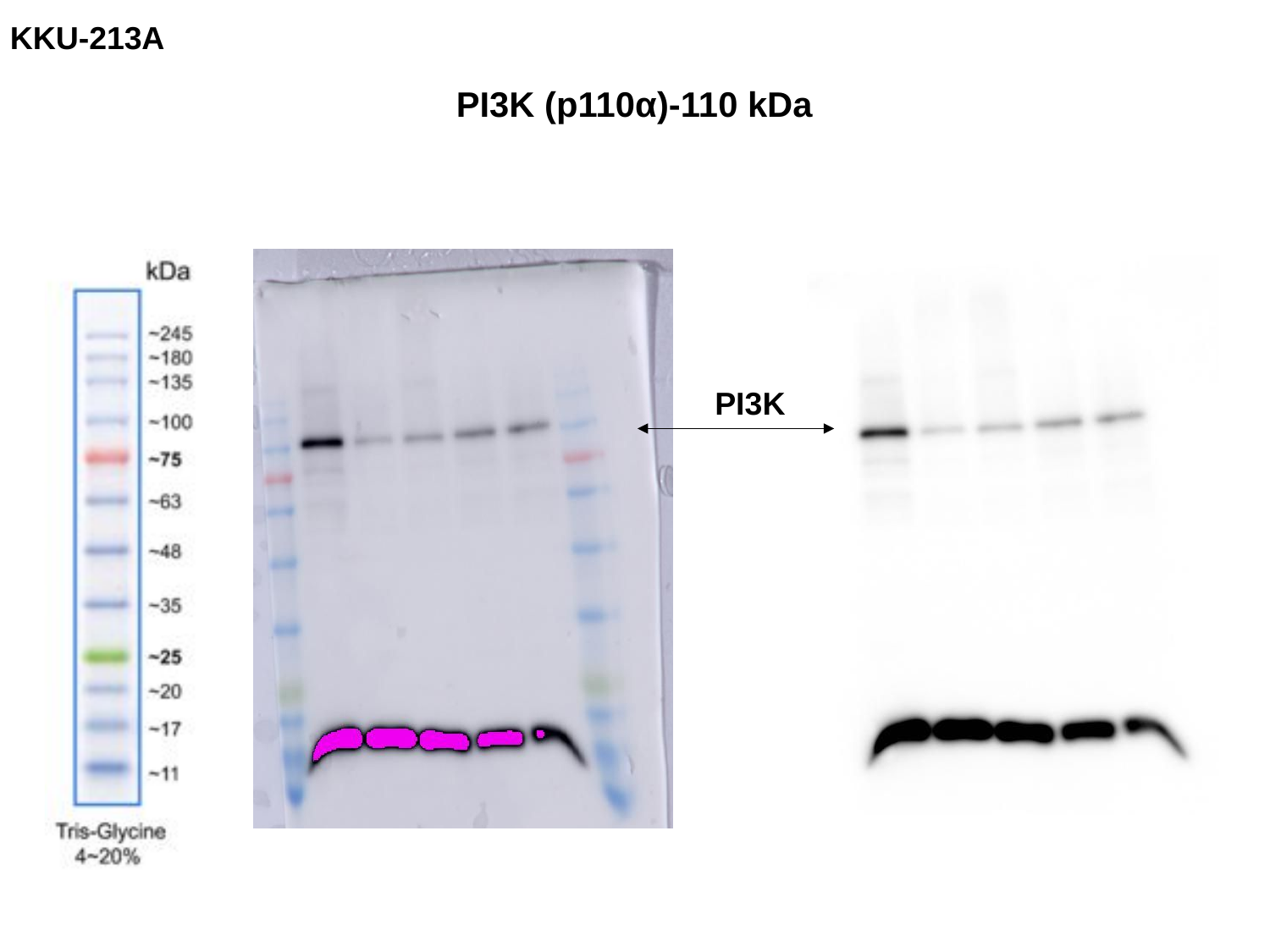

KKU-213A
PI3K (p110α)-110 kDa
PI3K

## Slide 6
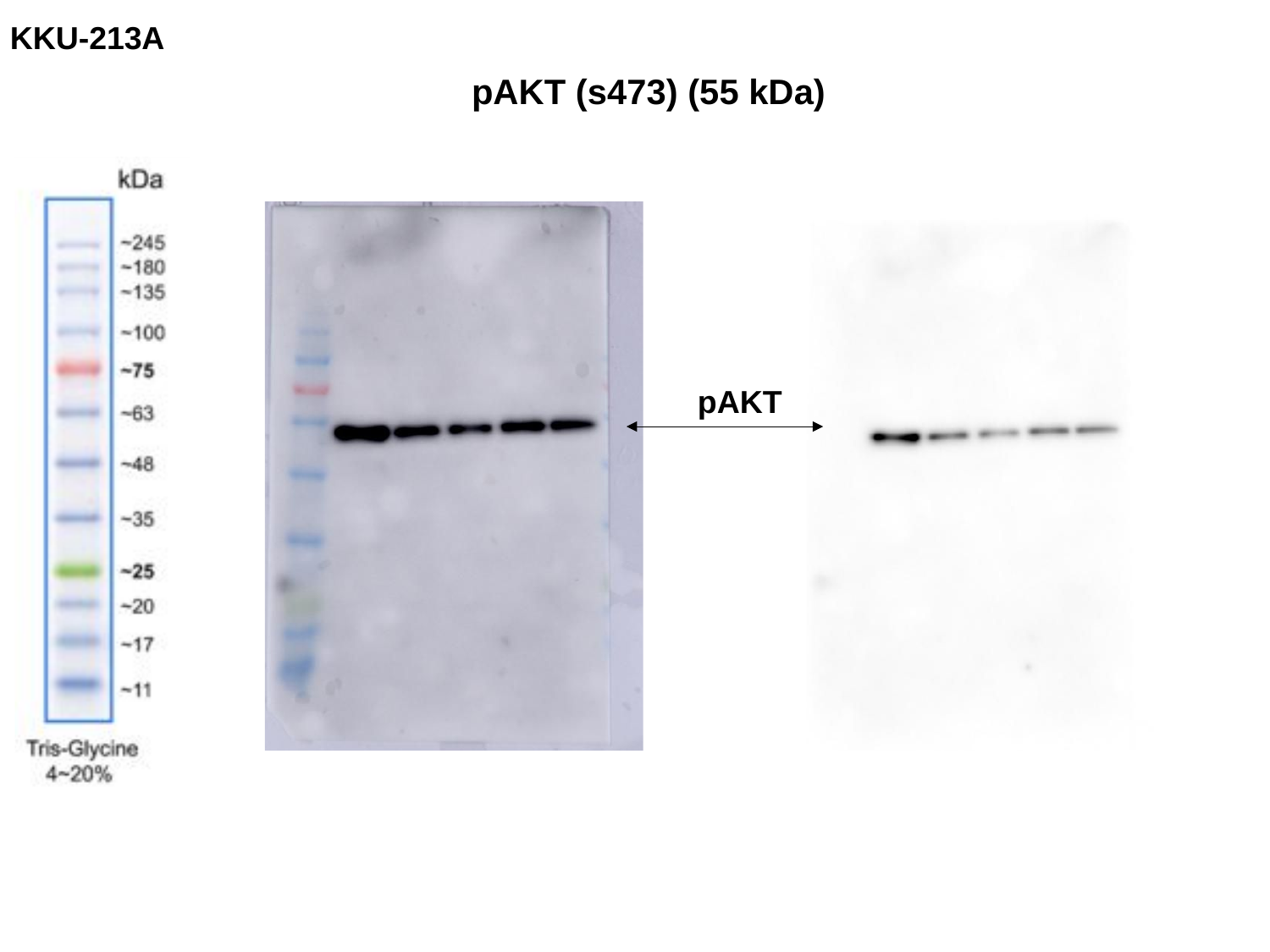

KKU-213A
pAKT (s473) (55 kDa)
pAKT

## Slide 7
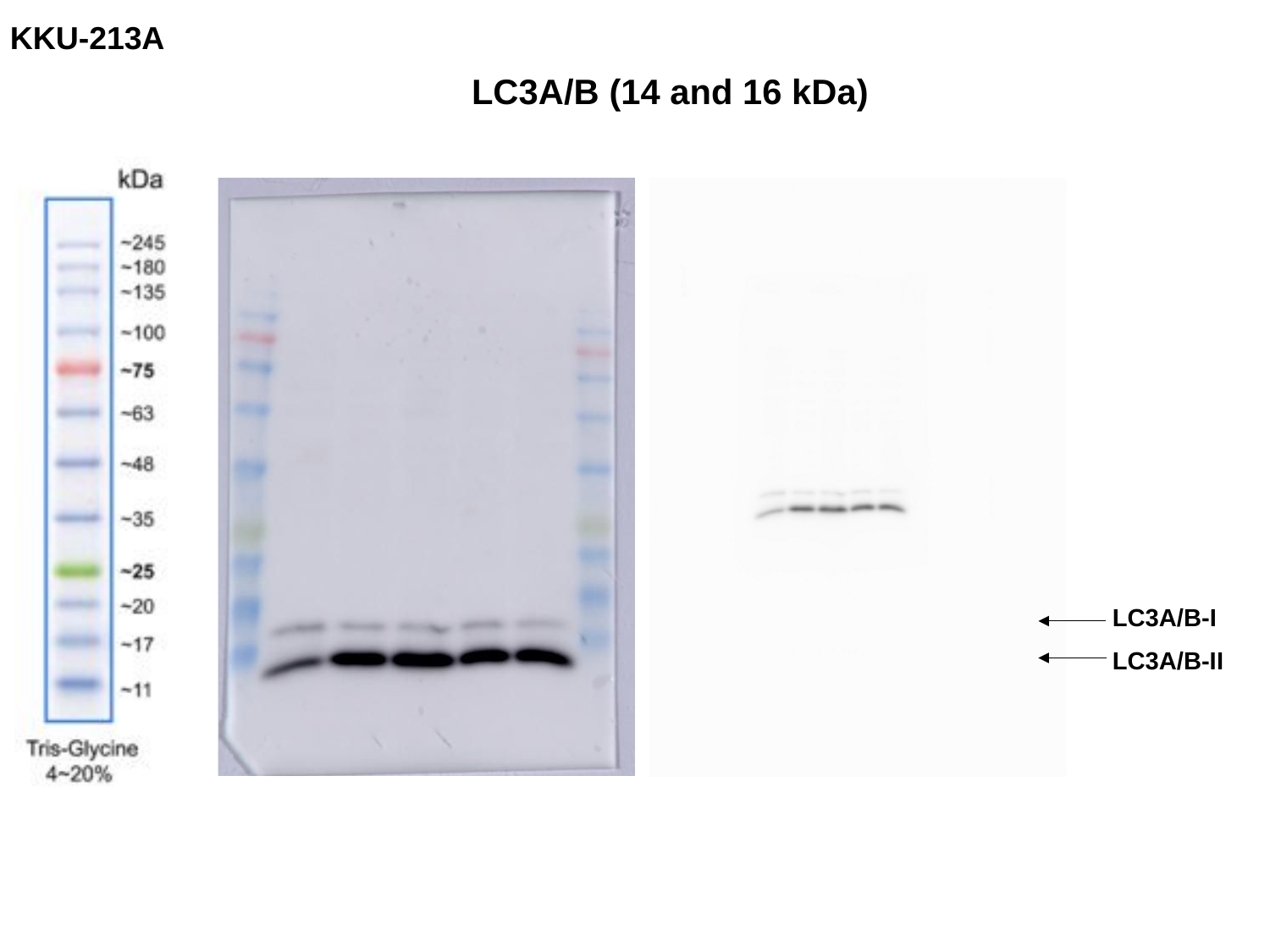

KKU-213A
LC3A/B (14 and 16 kDa)
LC3A/B-I
LC3A/B-II

## Slide 8
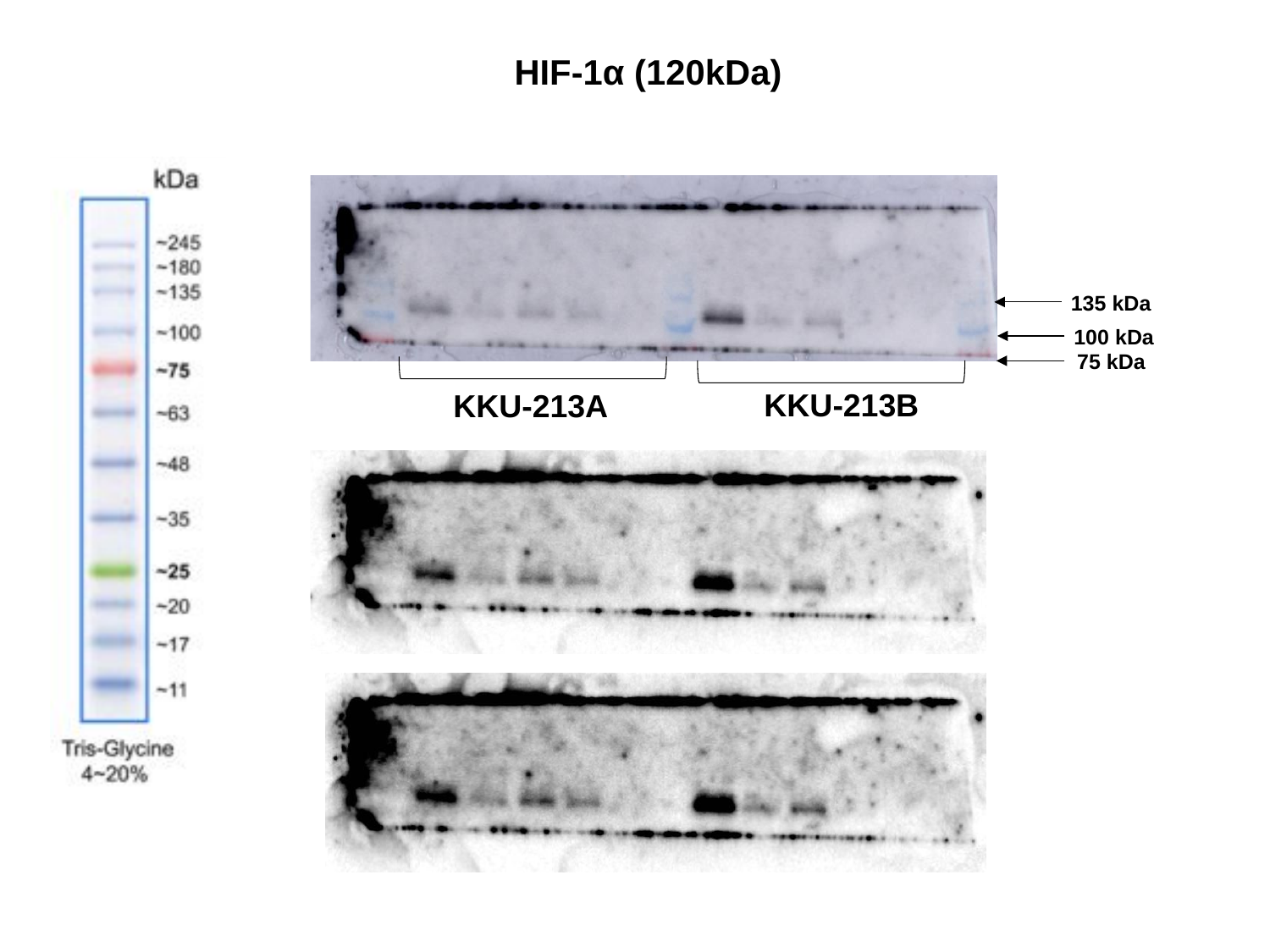

HIF-1α (120kDa)
135 kDa
100 kDa
75 kDa
KKU-213B
KKU-213A

## Slide 9
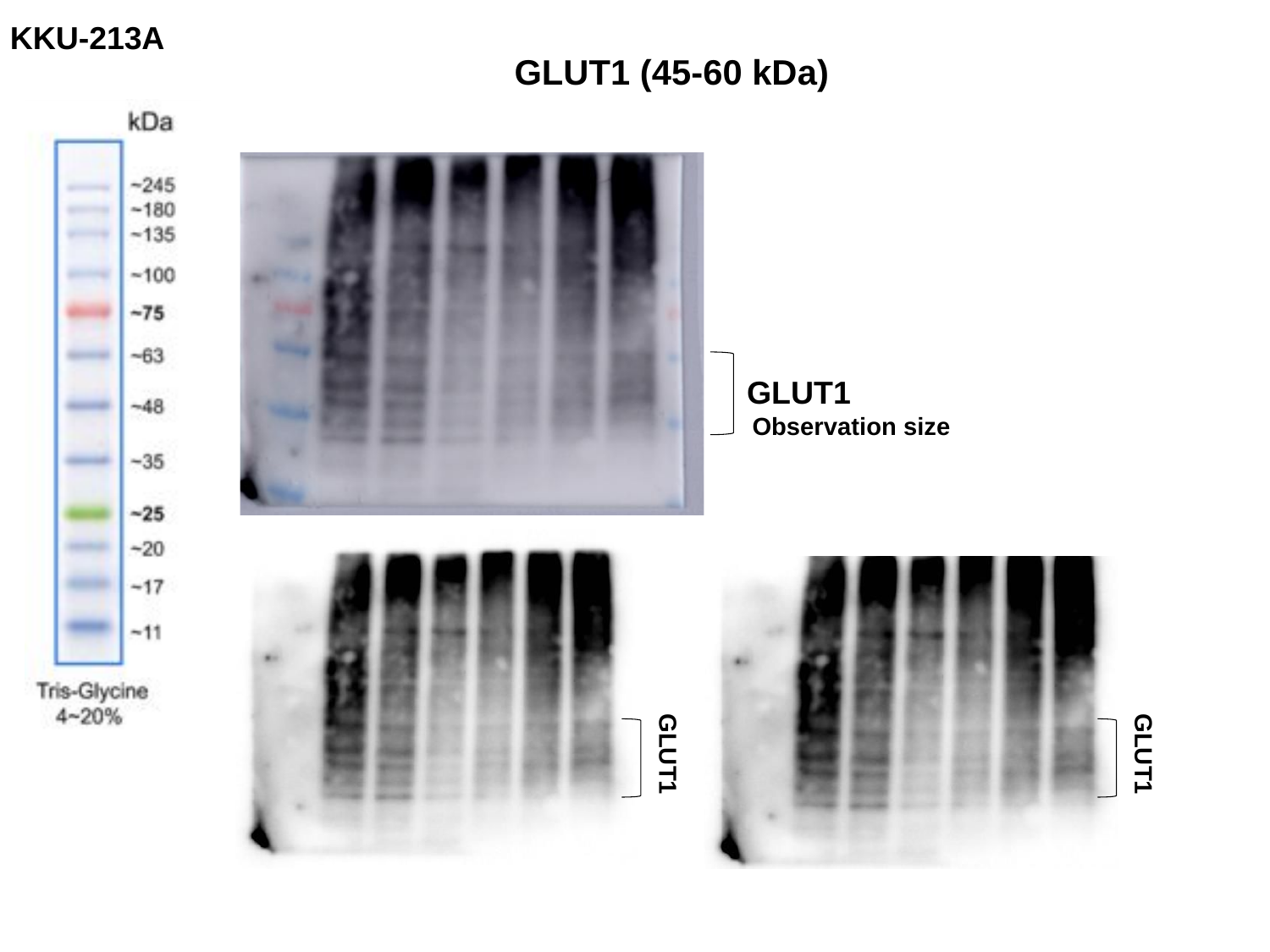

KKU-213A
GLUT1 (45-60 kDa)
GLUT1
Observation size
GLUT1
GLUT1

## Slide 10
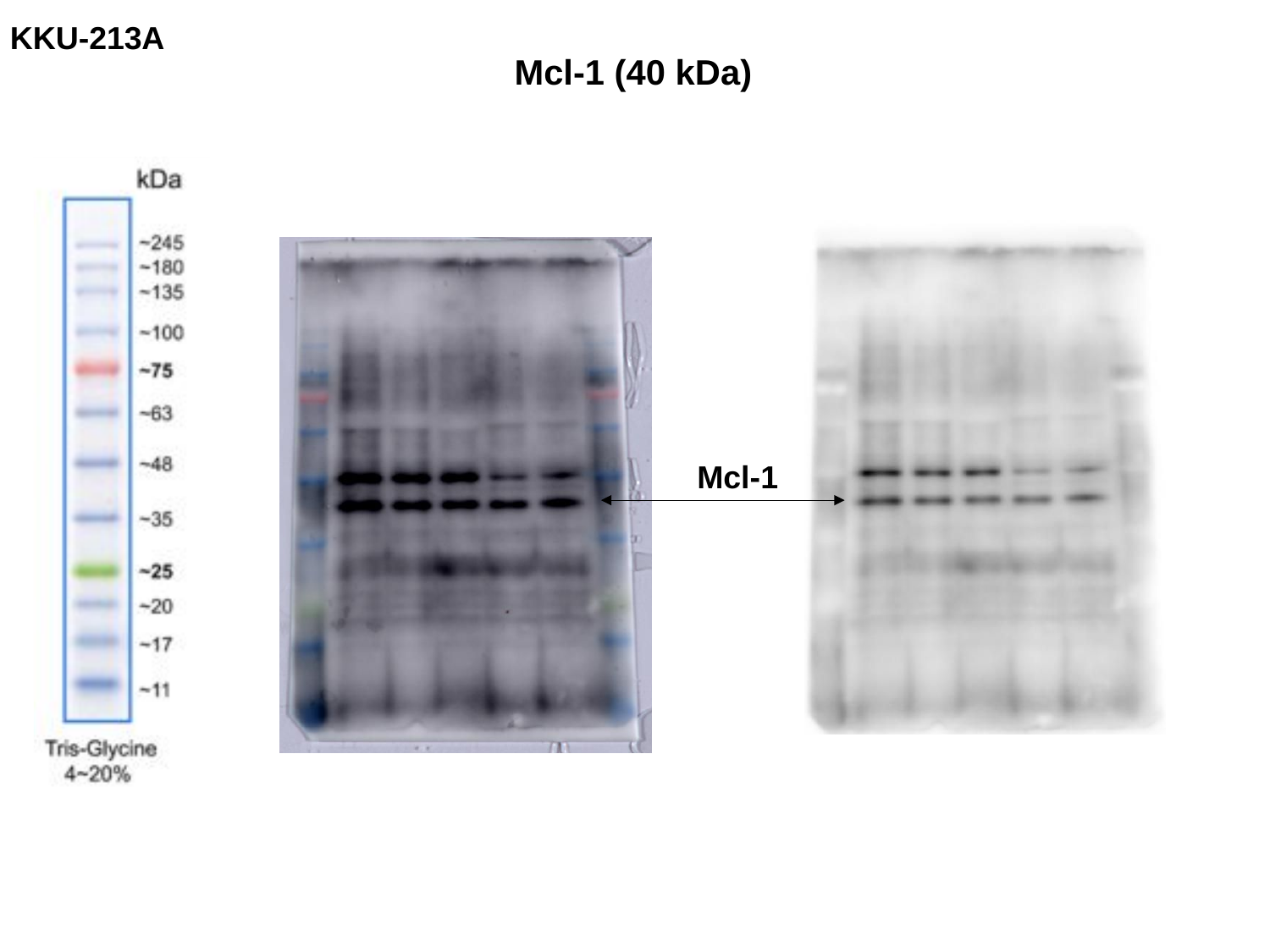

KKU-213A
Mcl-1 (40 kDa)
Mcl-1

## Slide 11
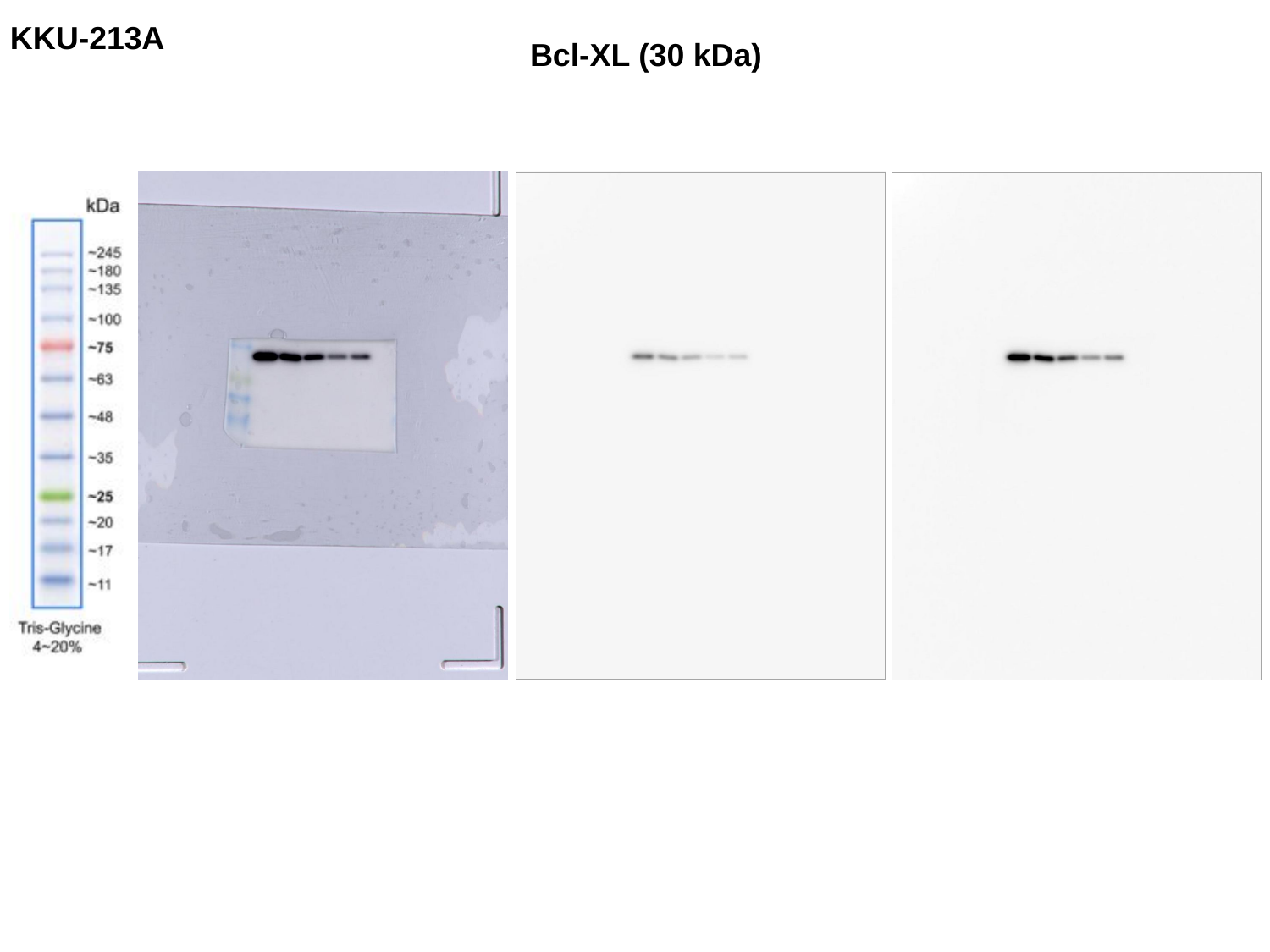

KKU-213A
Bcl-XL (30 kDa)

## Slide 12
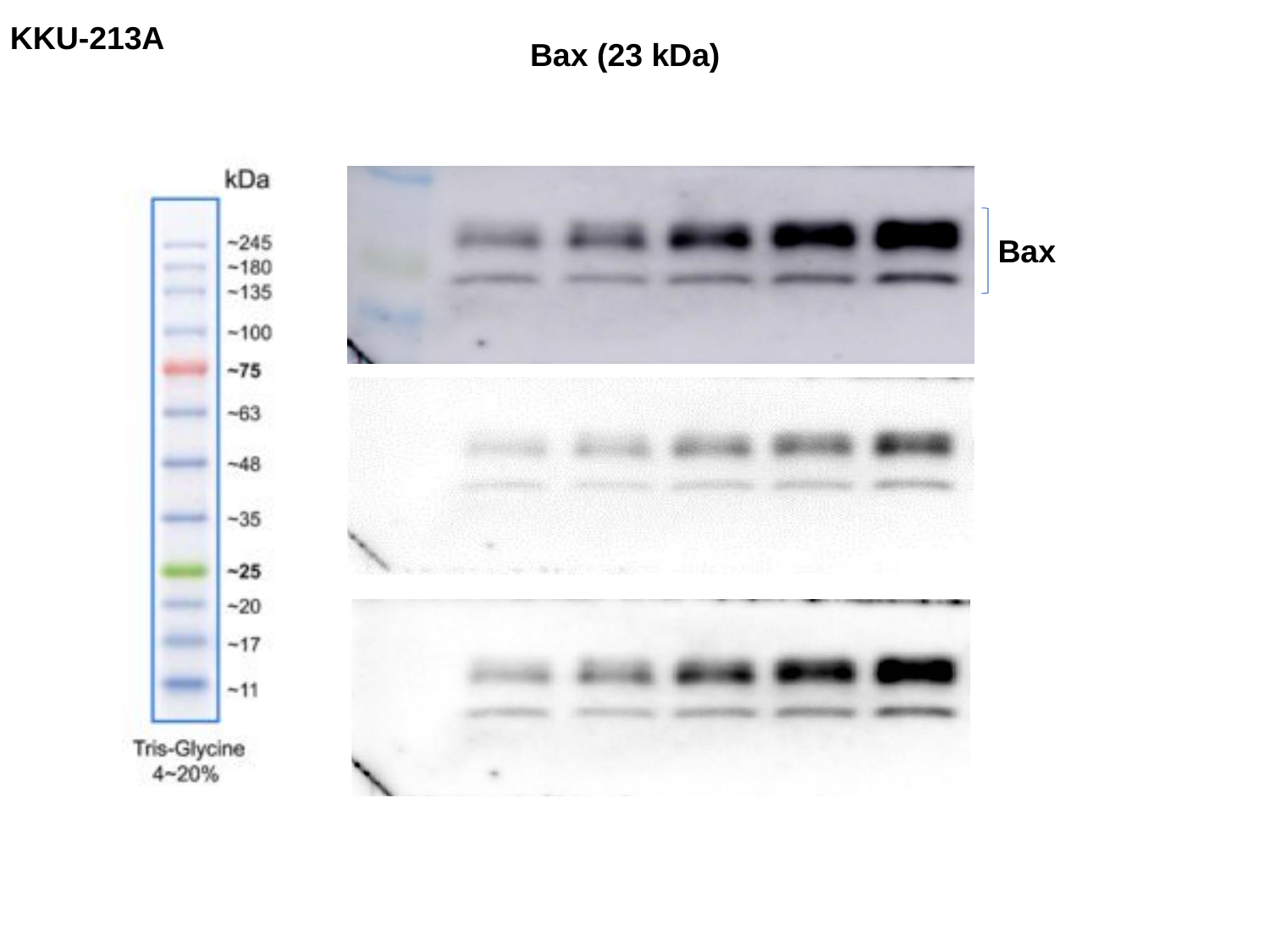

KKU-213A
Bax (23 kDa)
Bax

## Slide 13
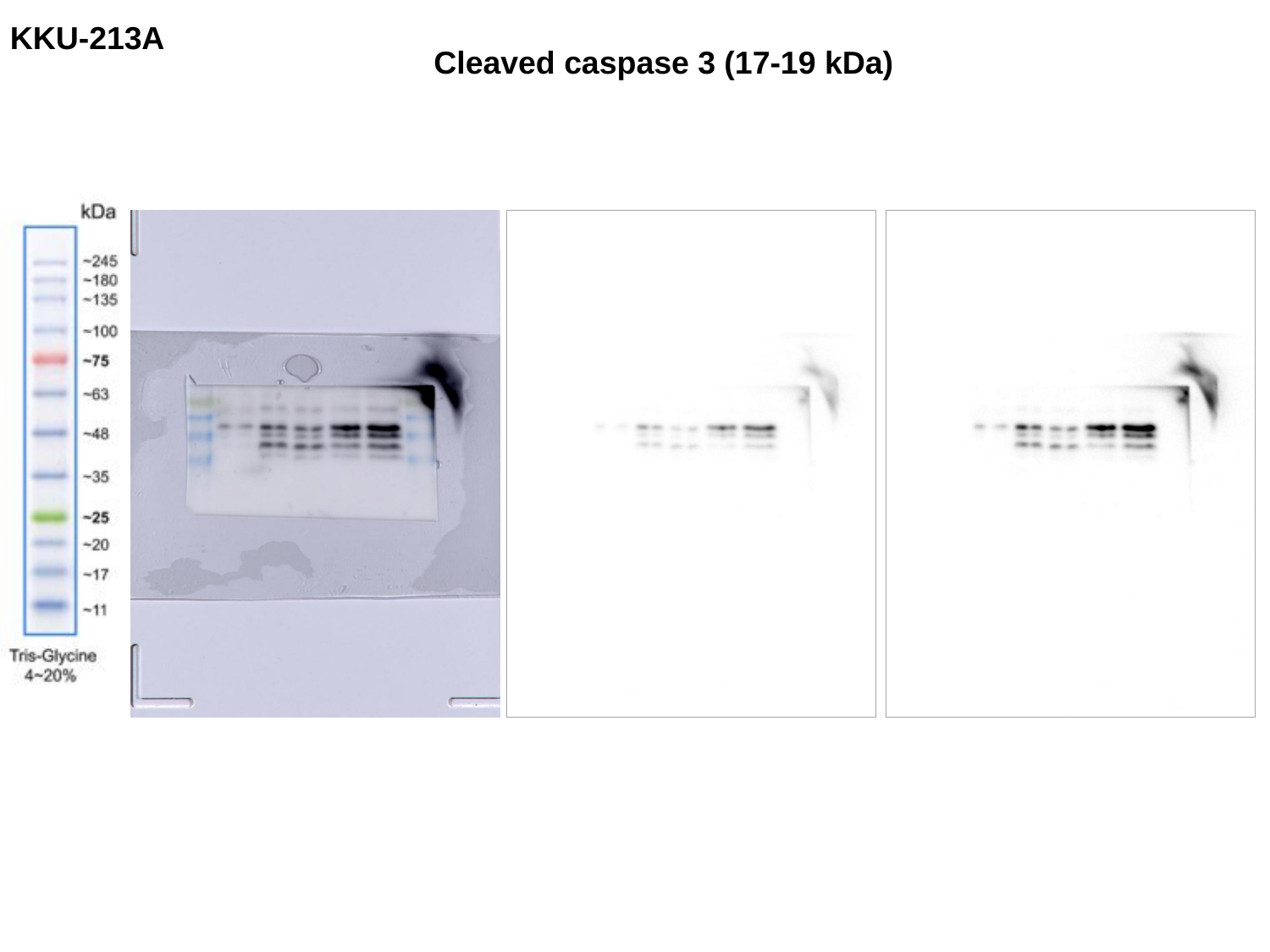

KKU-213A
Cleaved caspase 3 (17-19 kDa)

## Slide 14
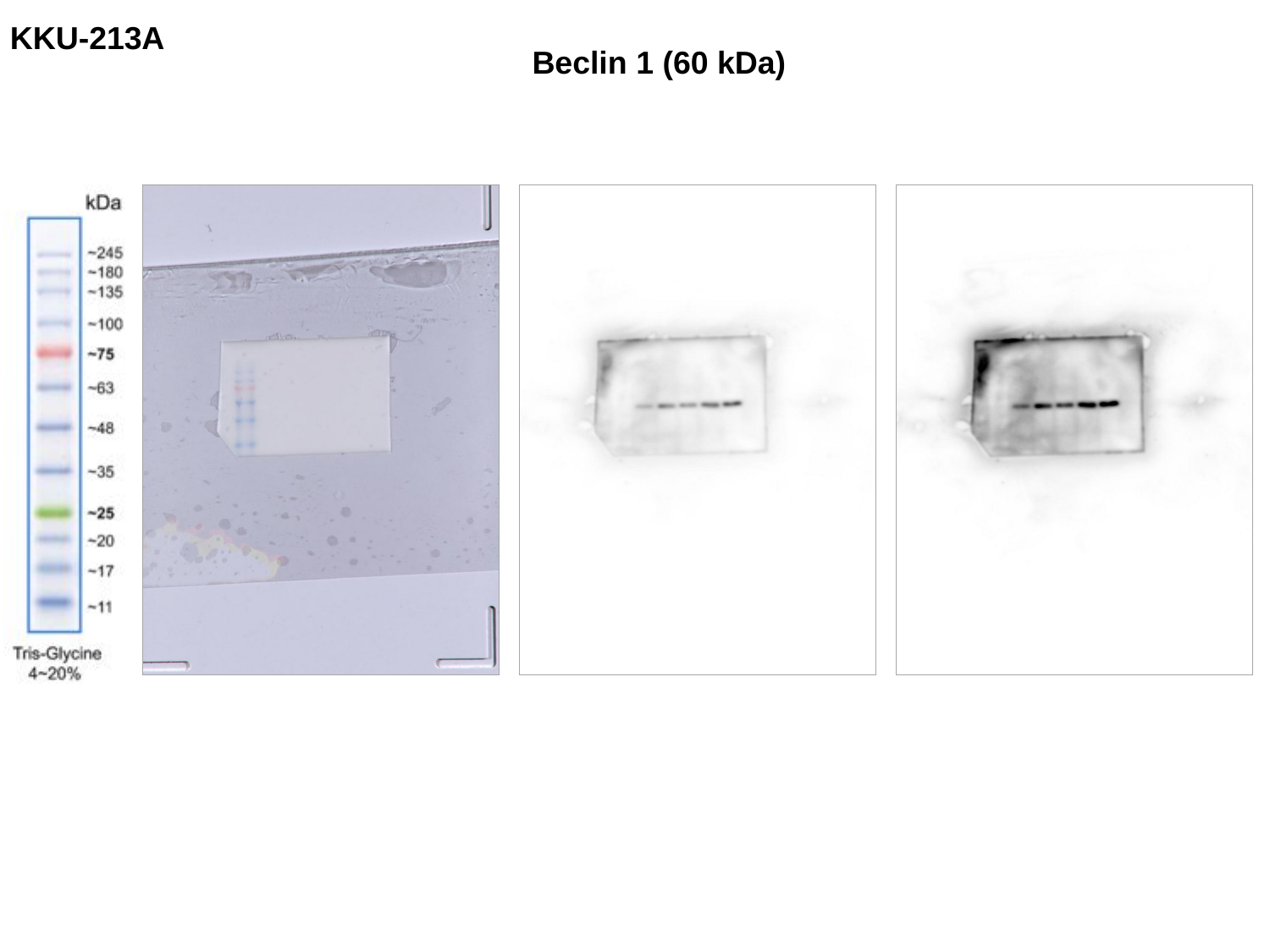

KKU-213A
Beclin 1 (60 kDa)

## Slide 15
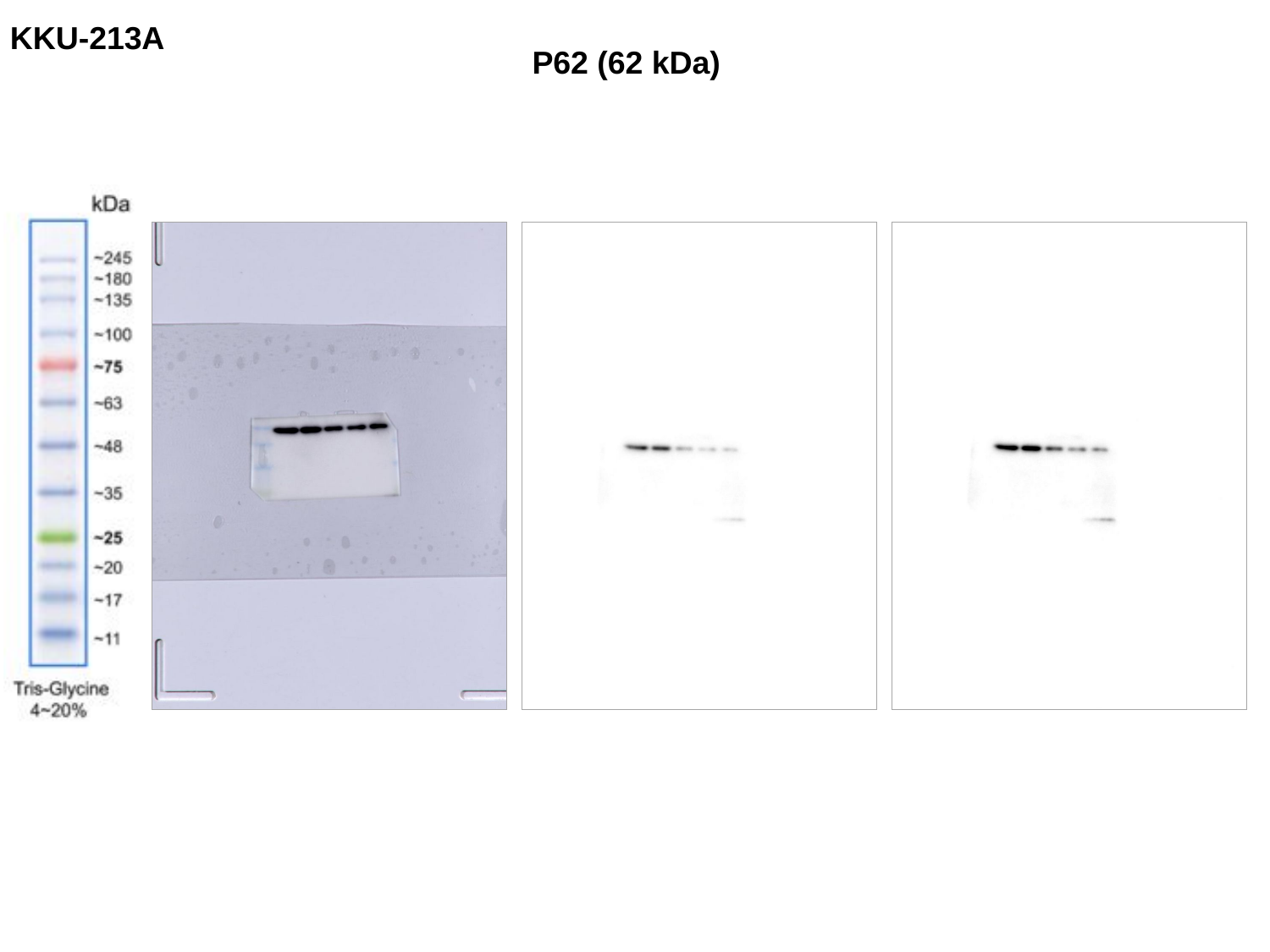

KKU-213A
P62 (62 kDa)

## Slide 16
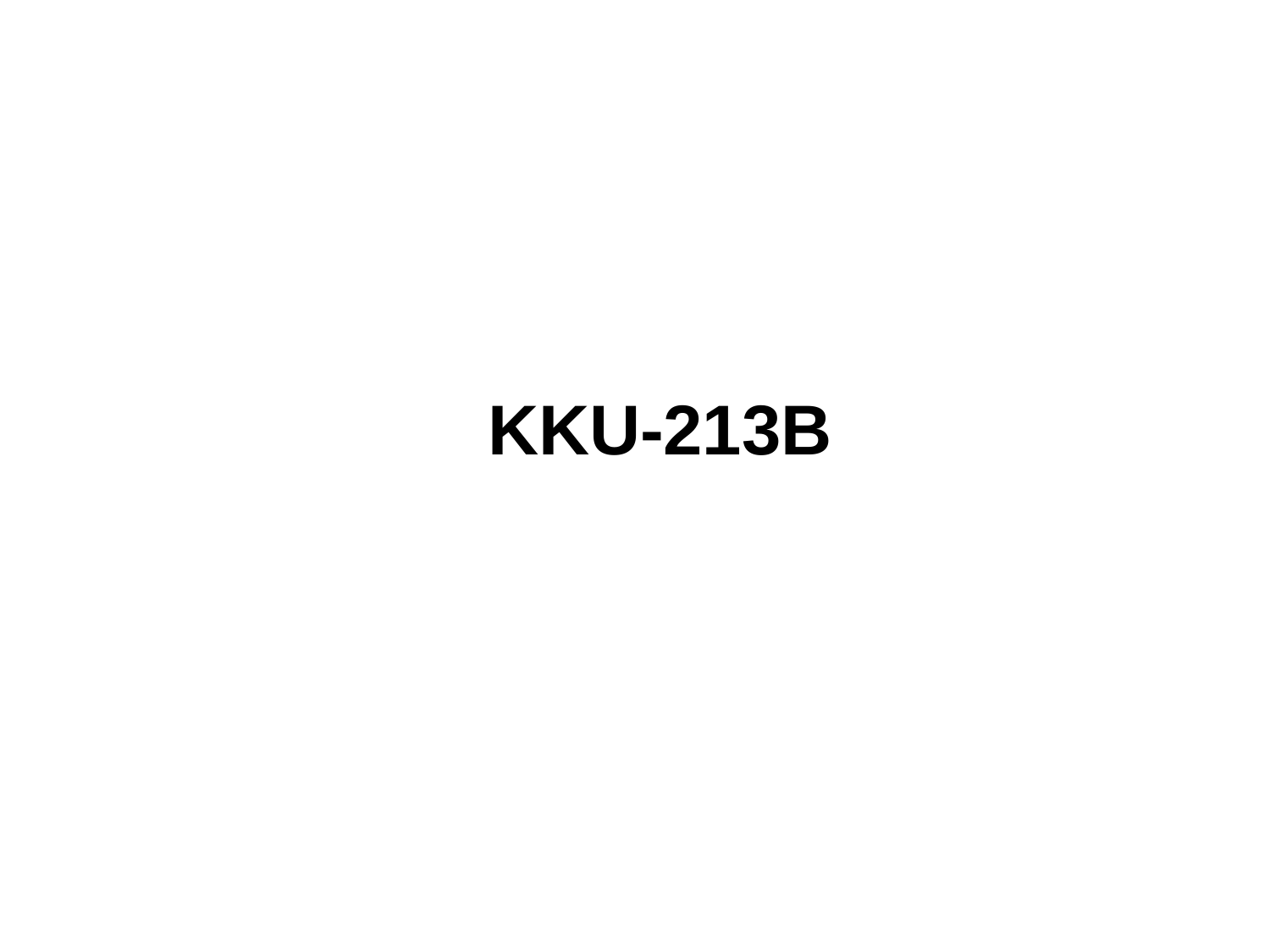

KKU-213B

## Slide 17
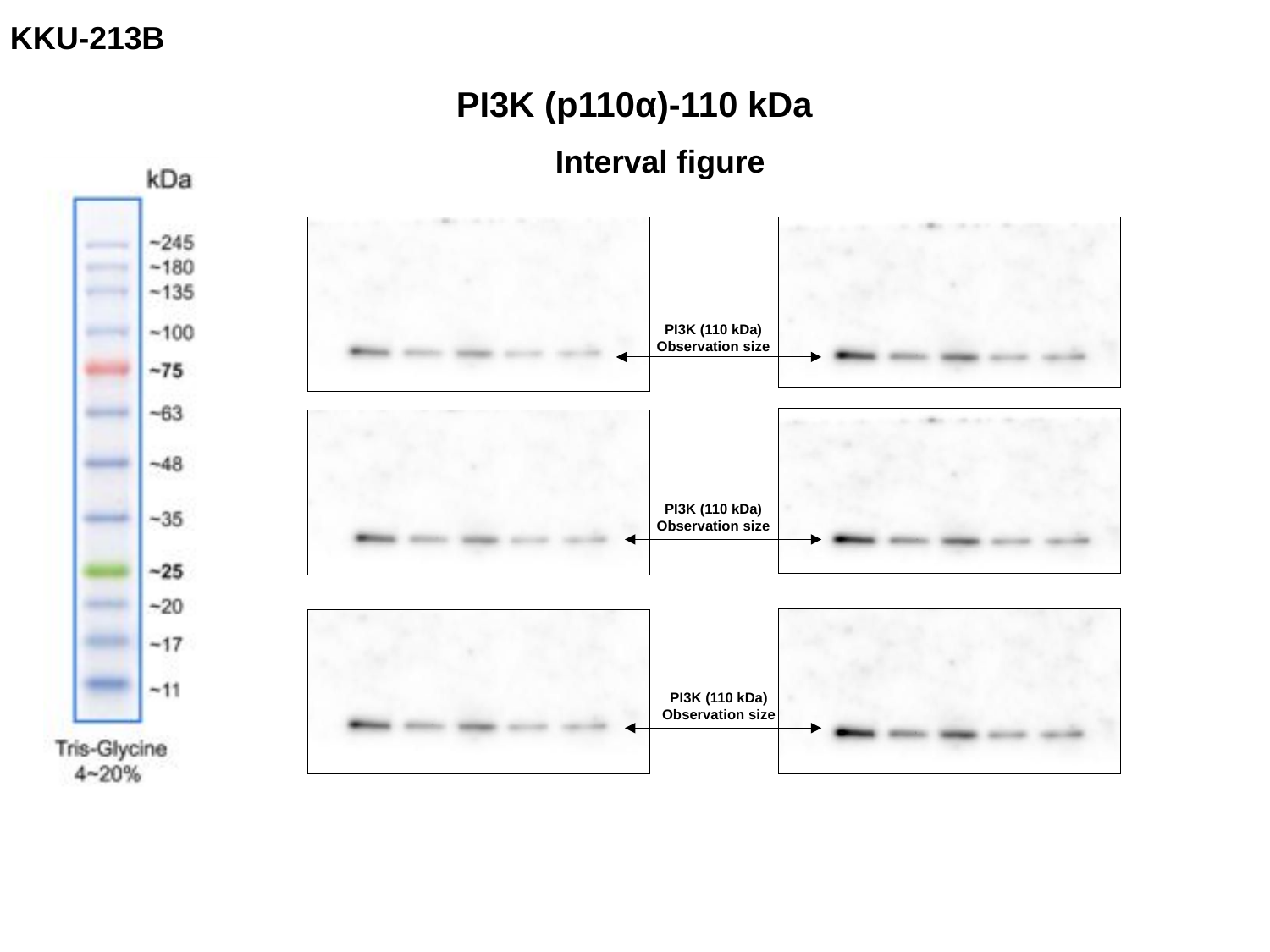

KKU-213B
PI3K (p110α)-110 kDa
Interval figure
PI3K (110 kDa)
Observation size
PI3K (110 kDa)
Observation size
PI3K (110 kDa)
Observation size

## Slide 18
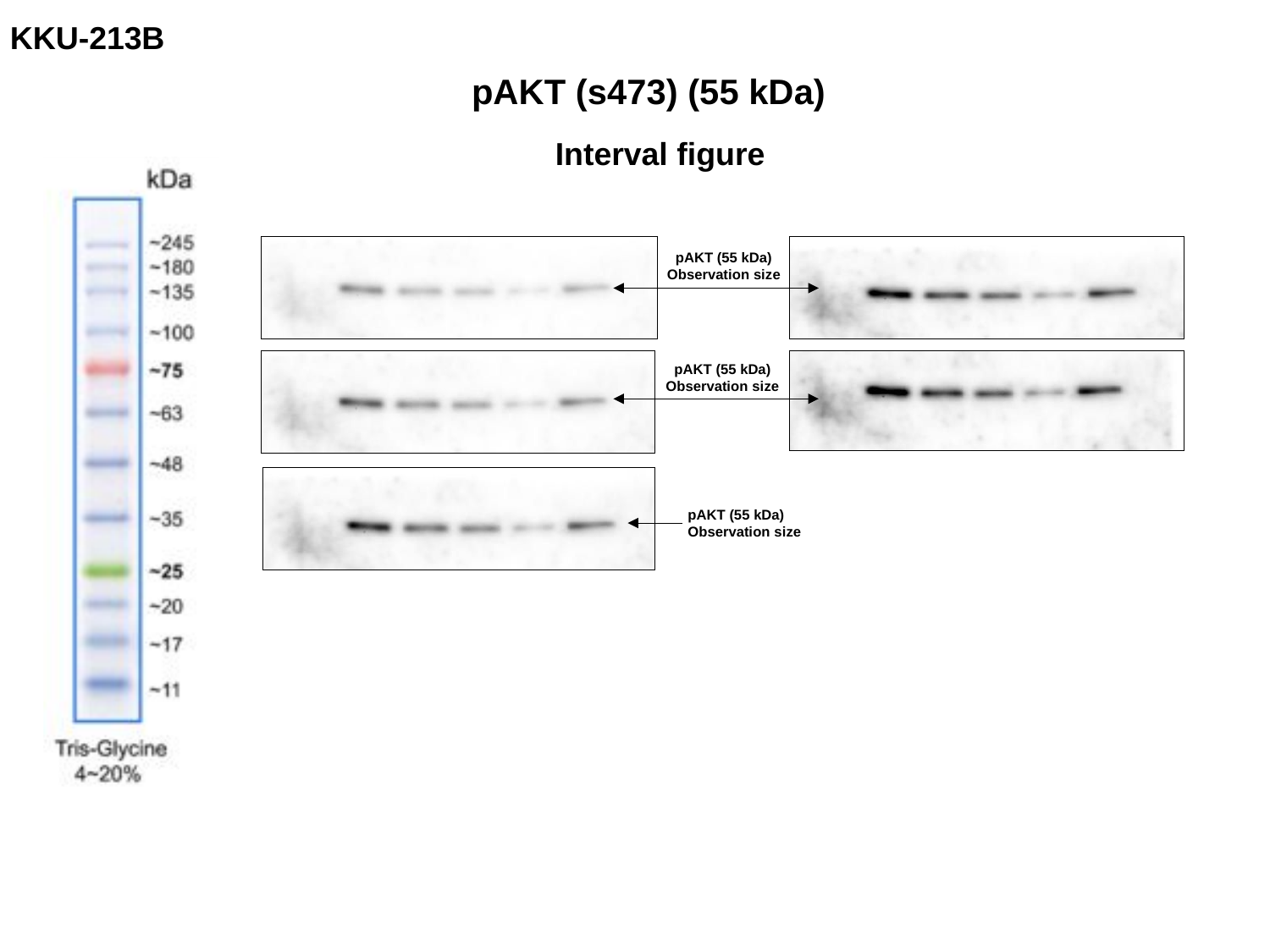

KKU-213B
pAKT (s473) (55 kDa)
Interval figure
pAKT (55 kDa)
Observation size
pAKT (55 kDa)
Observation size
pAKT (55 kDa)
Observation size

## Slide 19
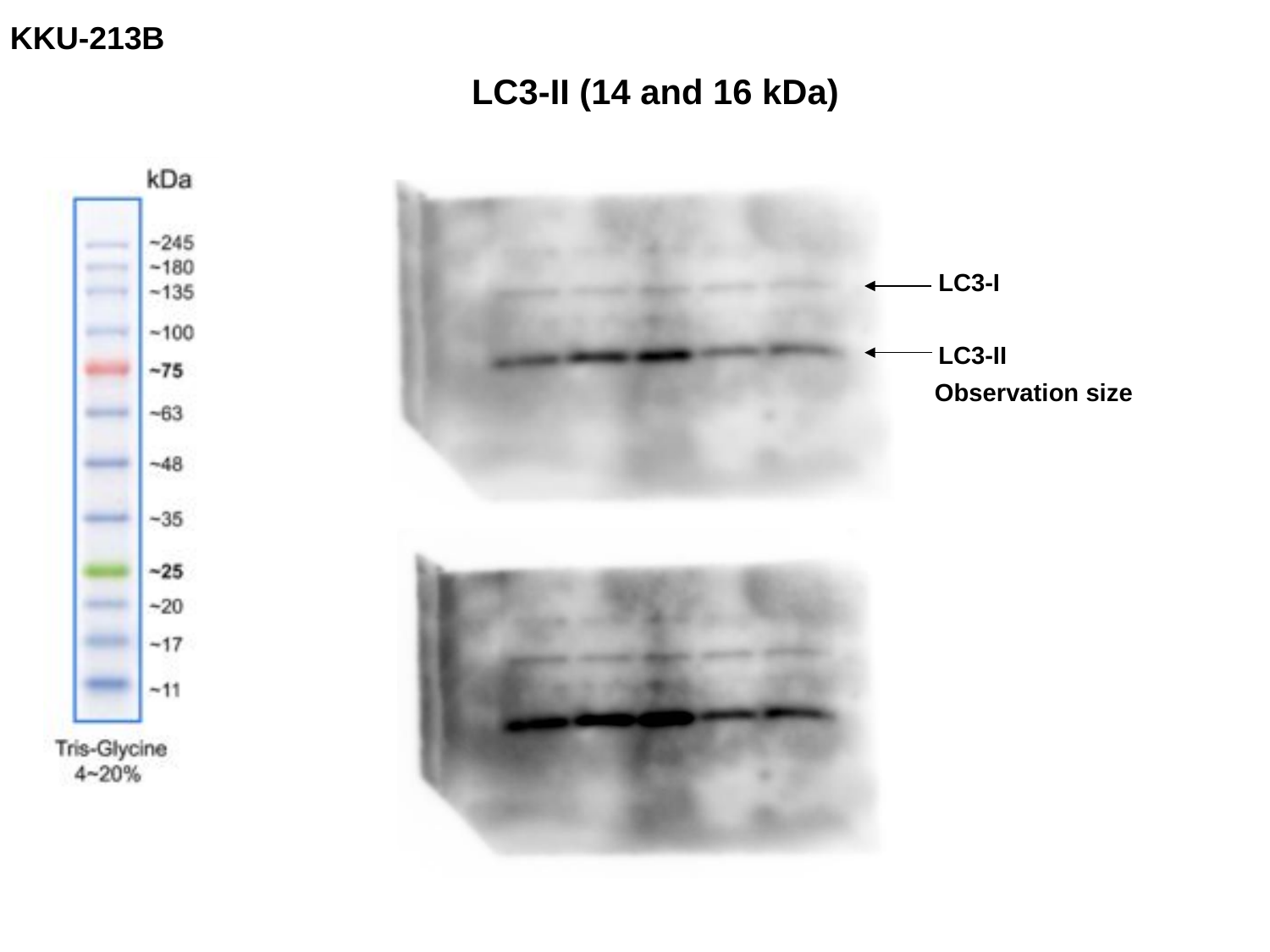

KKU-213B
LC3-II (14 and 16 kDa)
LC3-I
LC3-II
Observation size

## Slide 20
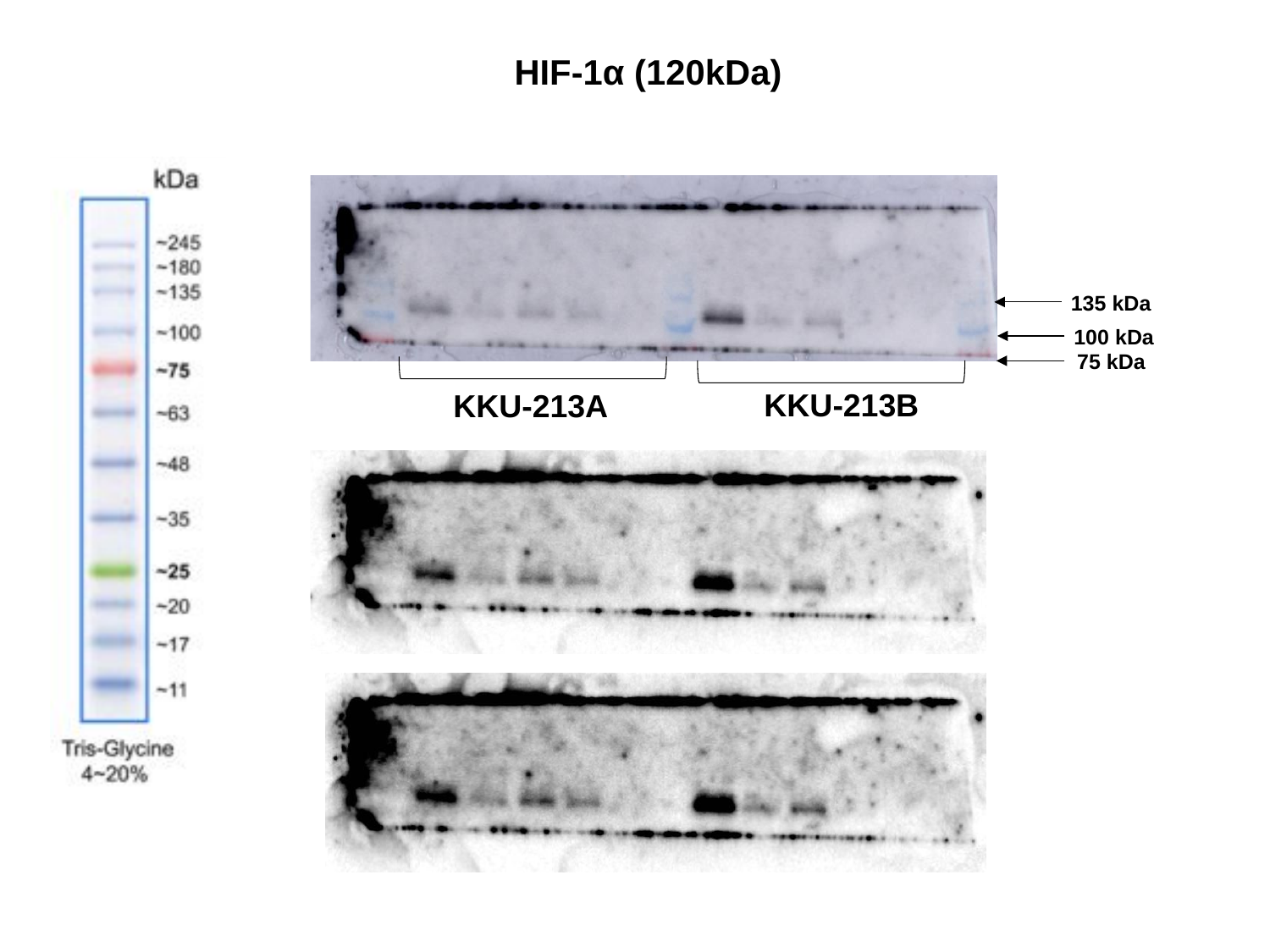

HIF-1α (120kDa)
135 kDa
100 kDa
75 kDa
KKU-213B
KKU-213A

## Slide 21
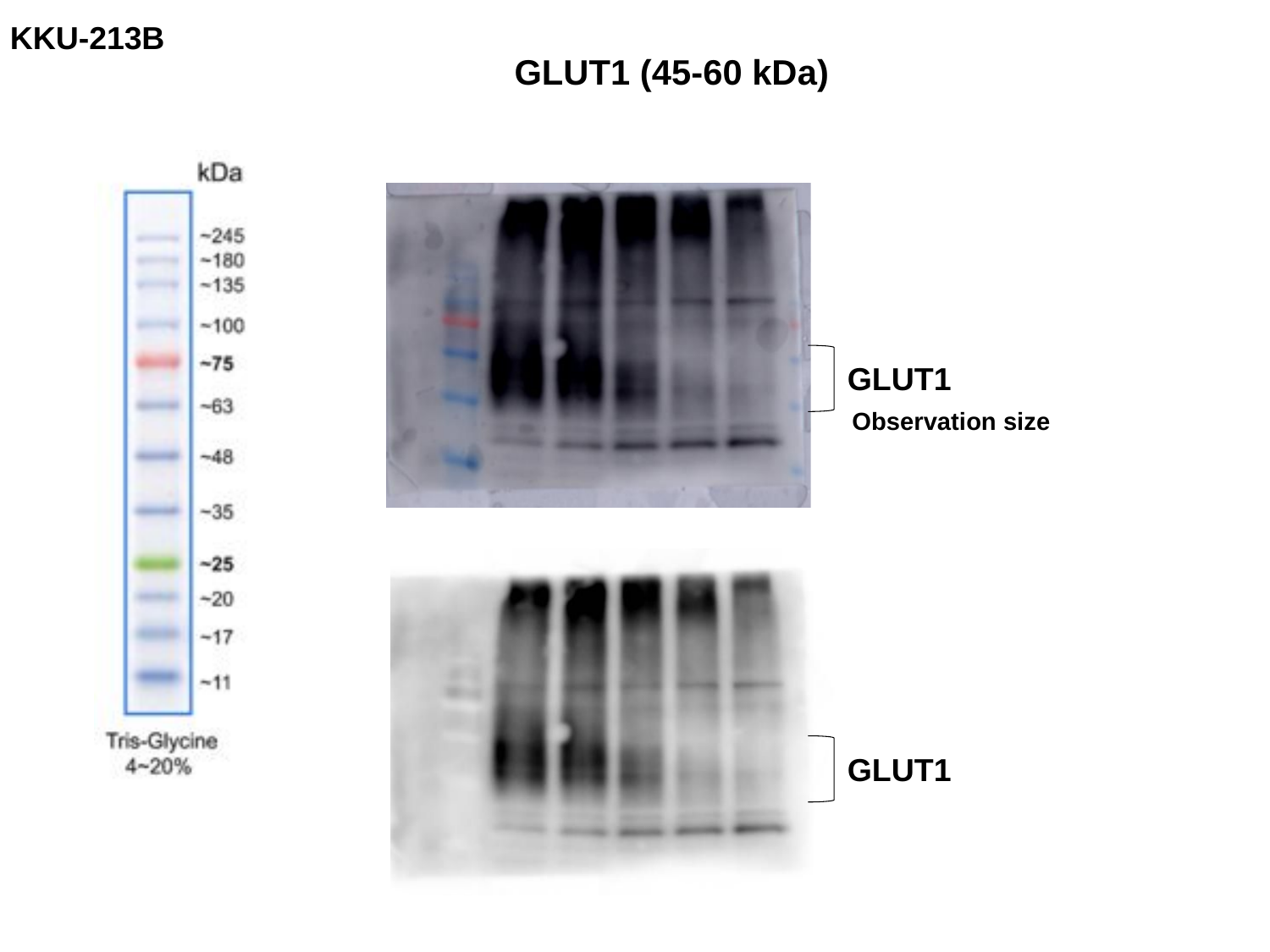

KKU-213B
GLUT1 (45-60 kDa)
GLUT1
Observation size
GLUT1

## Slide 22
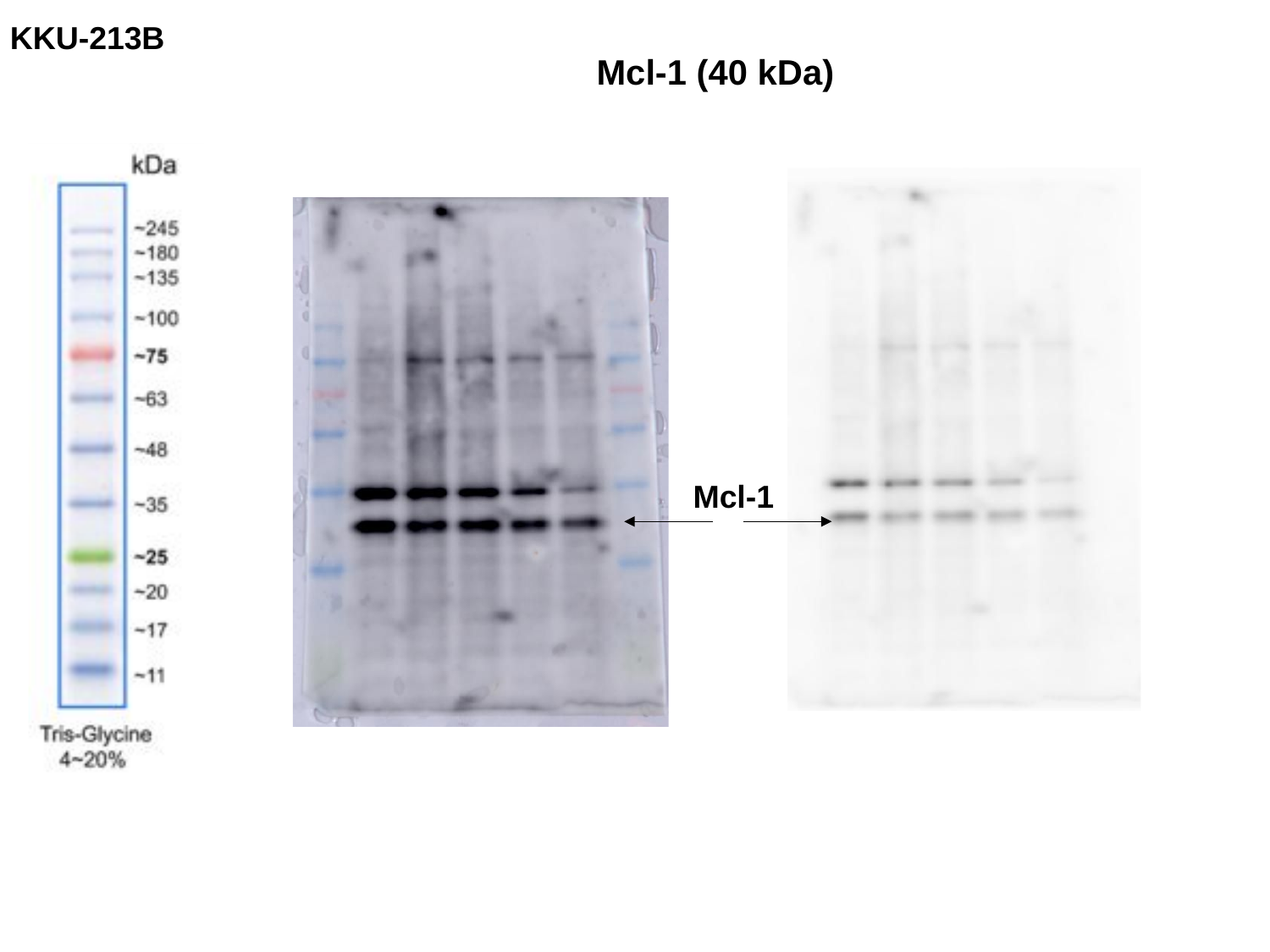

KKU-213B
Mcl-1 (40 kDa)
Mcl-1

## Slide 23
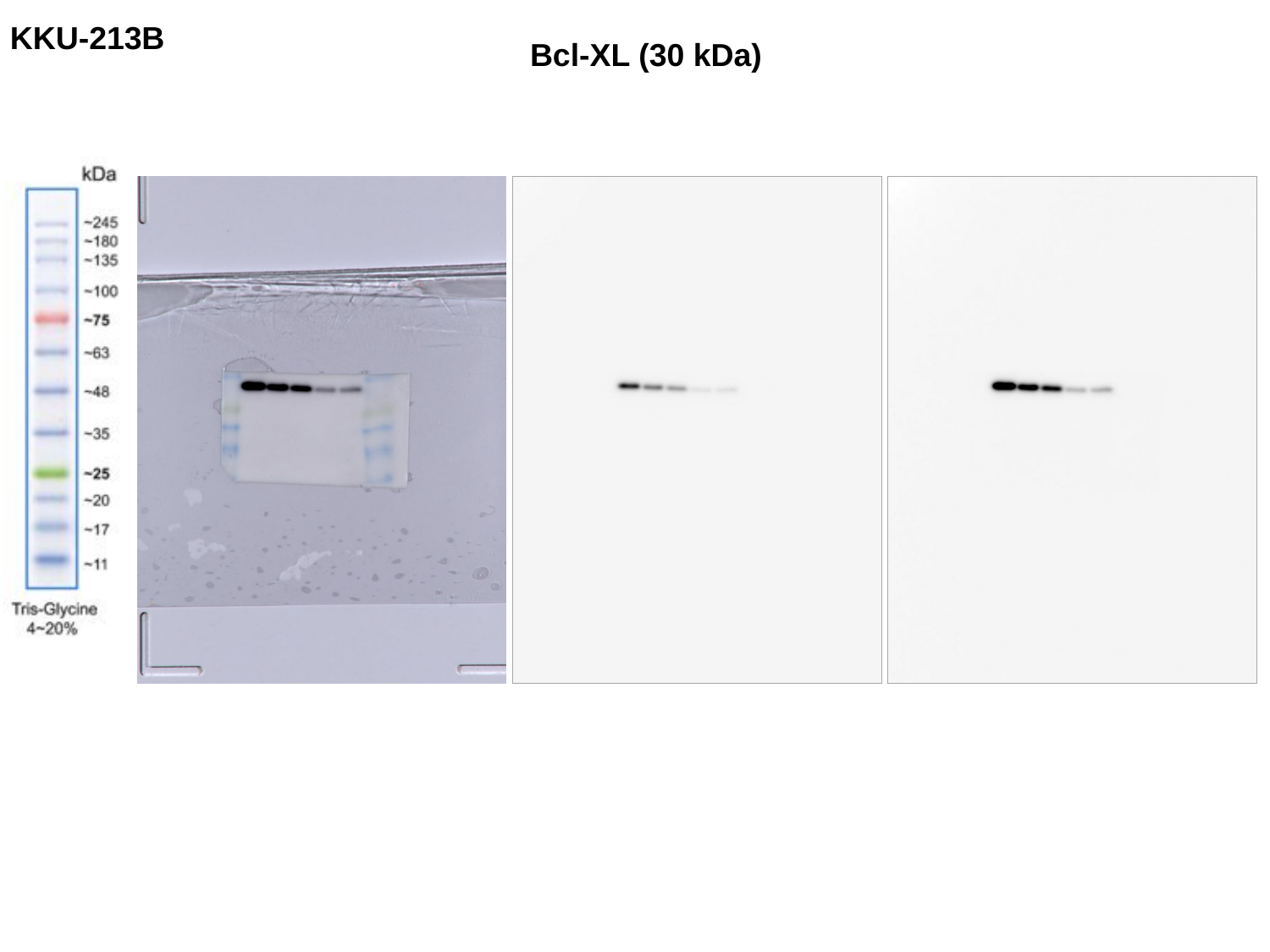

KKU-213B
Bcl-XL (30 kDa)

## Slide 24
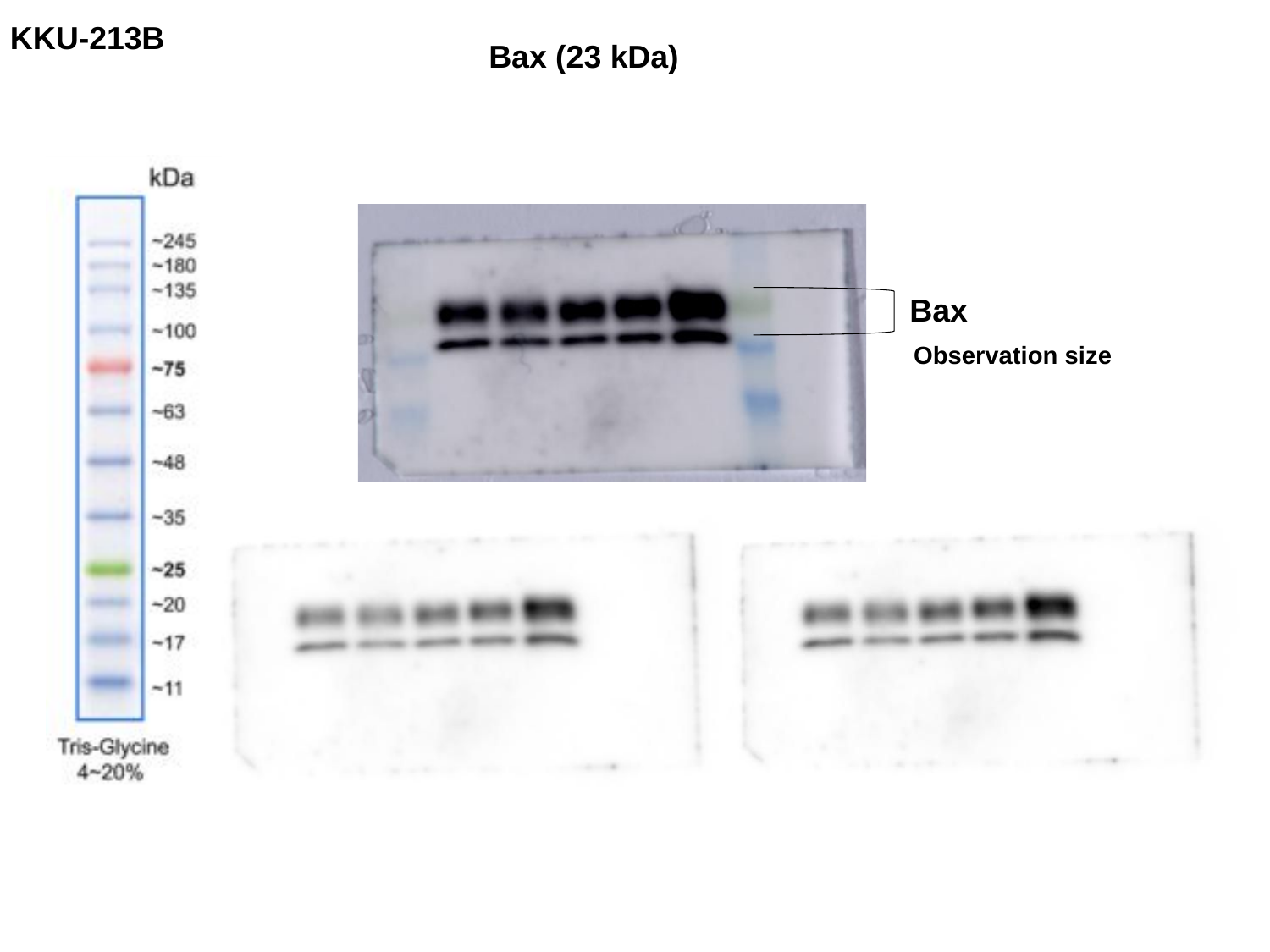

KKU-213B
Bax (23 kDa)
Bax
Observation size

## Slide 25
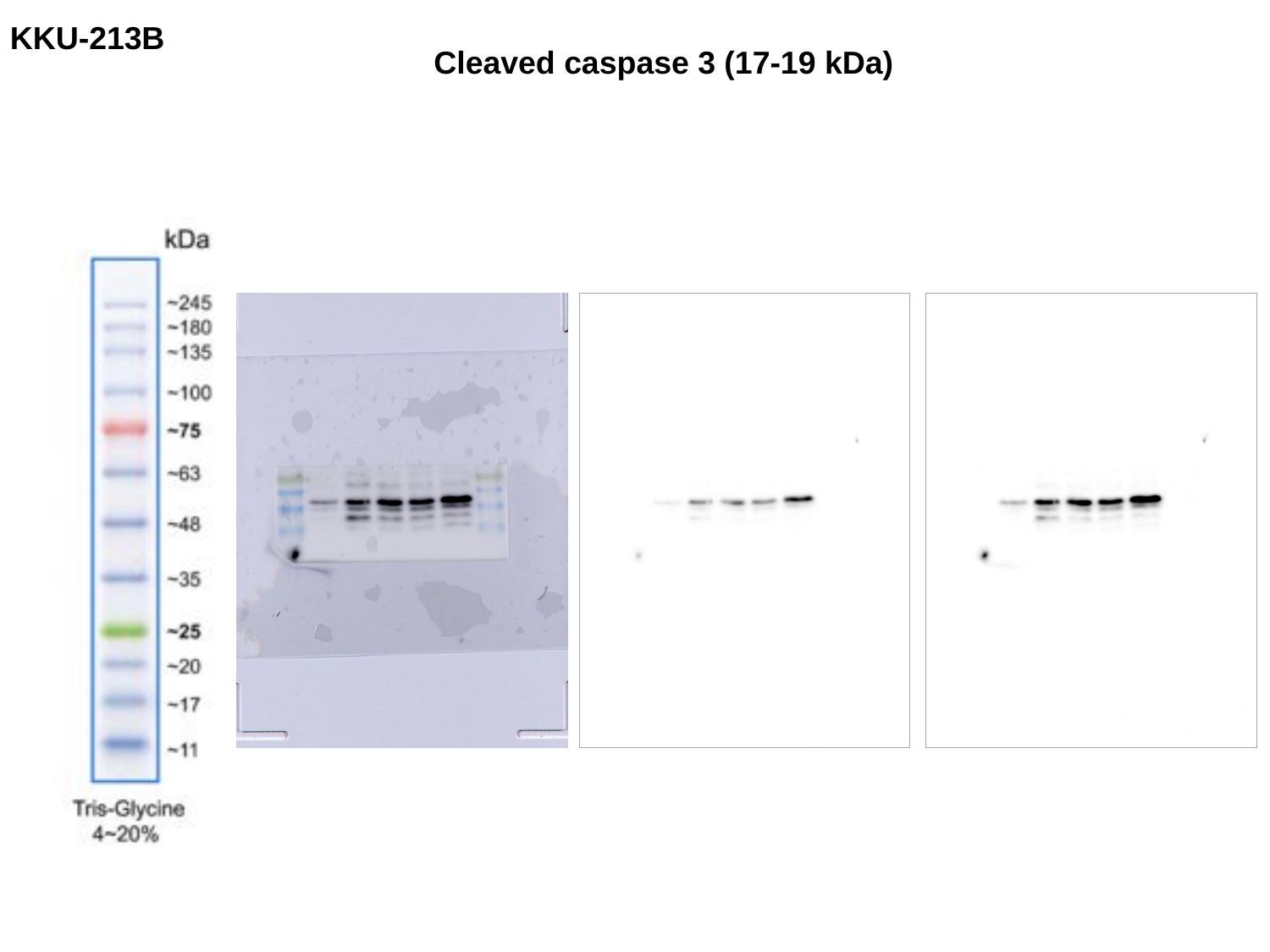

KKU-213B
Cleaved caspase 3 (17-19 kDa)

## Slide 26
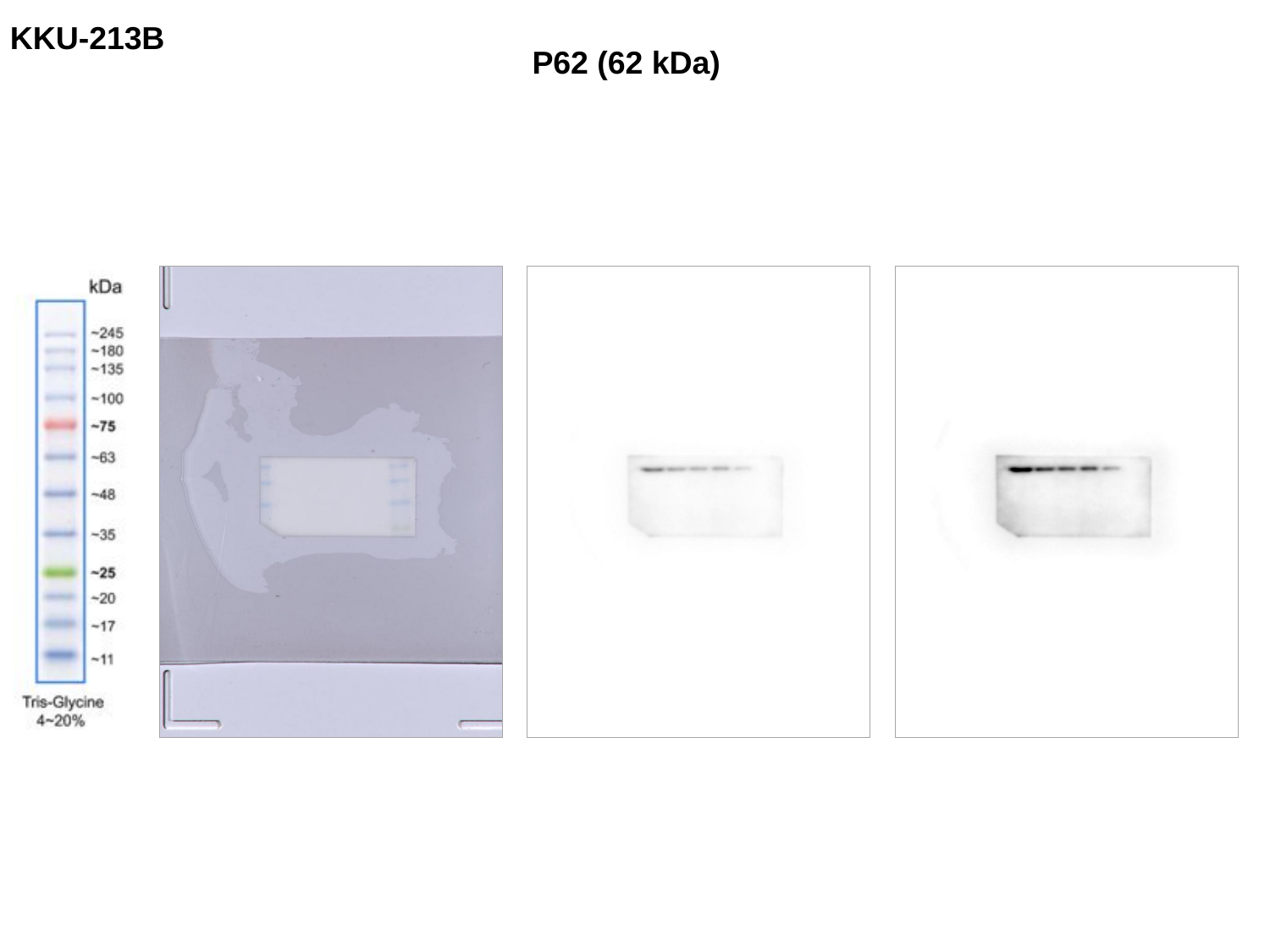

KKU-213B
P62 (62 kDa)

## Slide 27
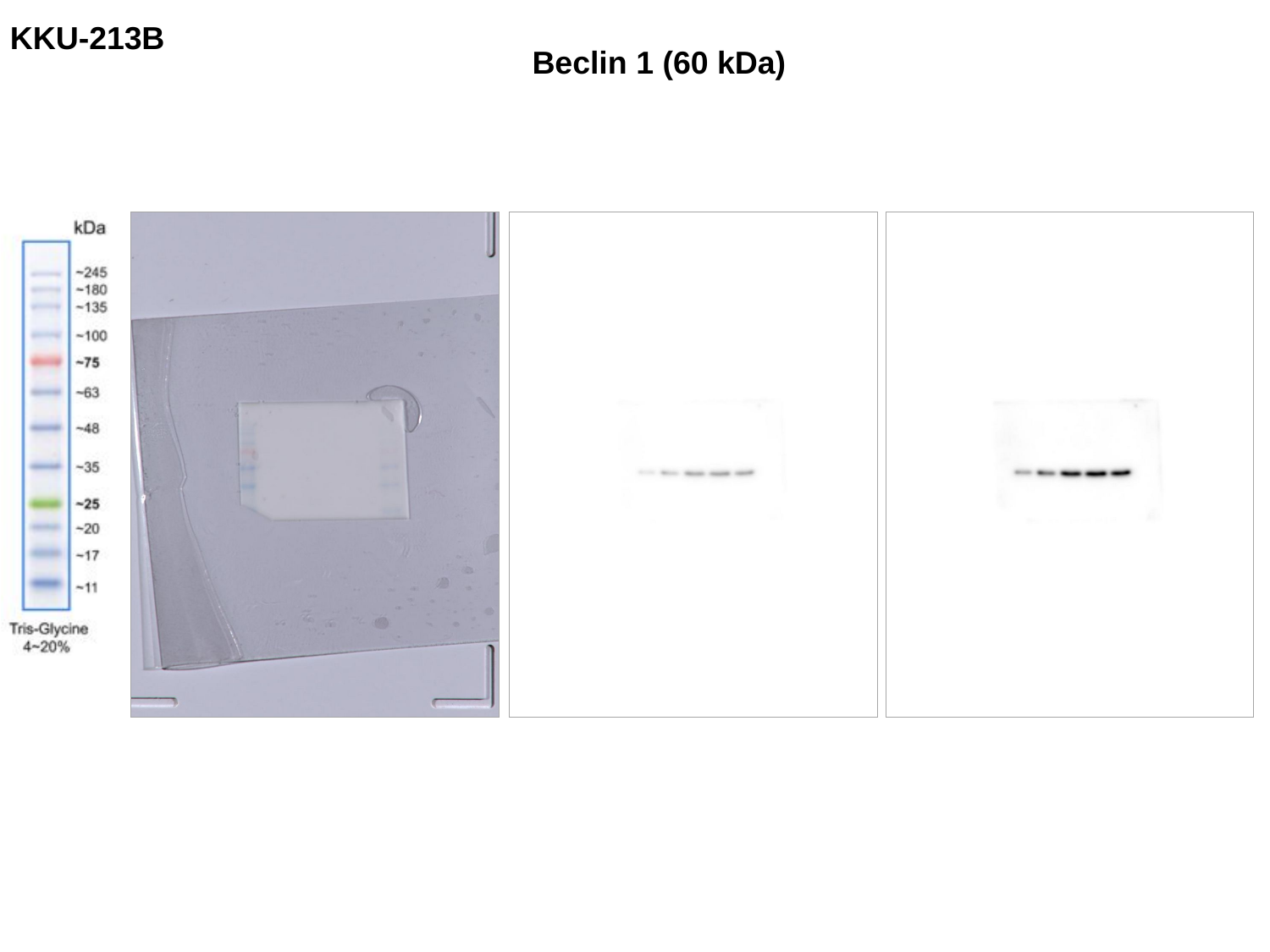

KKU-213B
Beclin 1 (60 kDa)

## Slide 28
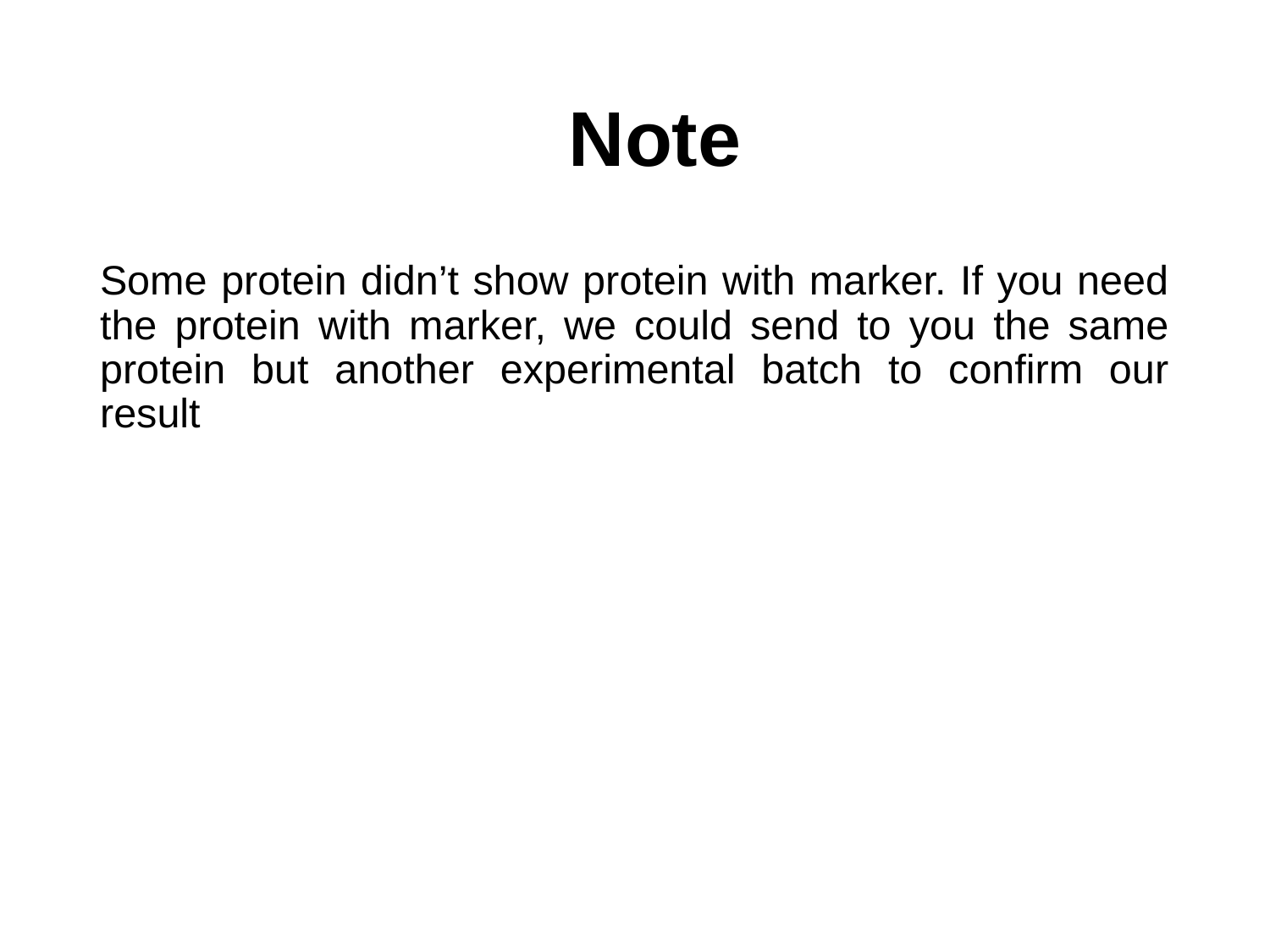

# Note
Some protein didn’t show protein with marker. If you need the protein with marker, we could send to you the same protein but another experimental batch to confirm our result
