# Supplementary material for: Reversine, a selective MPS1 inhibitor, induced autophagic cell death via diminished glucose uptake and ATP production in cholangiocarcinoma cells
Source: PeerJ. 2021 Jan 7;9:e10637. doi: 10.7717/peerj.10637 (PMC7797171; doi:10.7717/peerj.10637)
Supplement: Supplemental Information 6 [file peerj-09-10637-s006.zip › Original figure 4.pptx]

## Slide 1
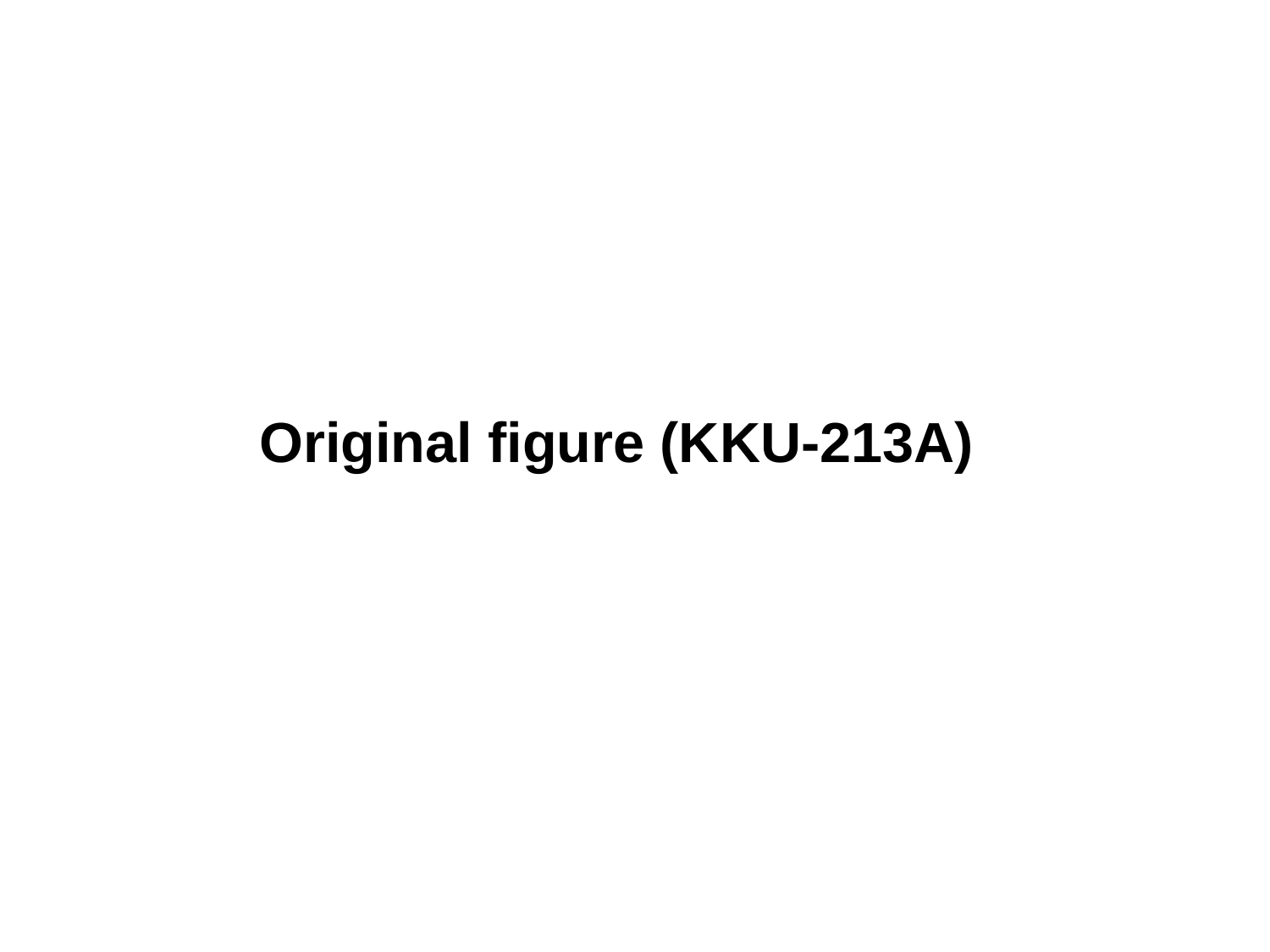

Original figure (KKU-213A)

## Slide 2
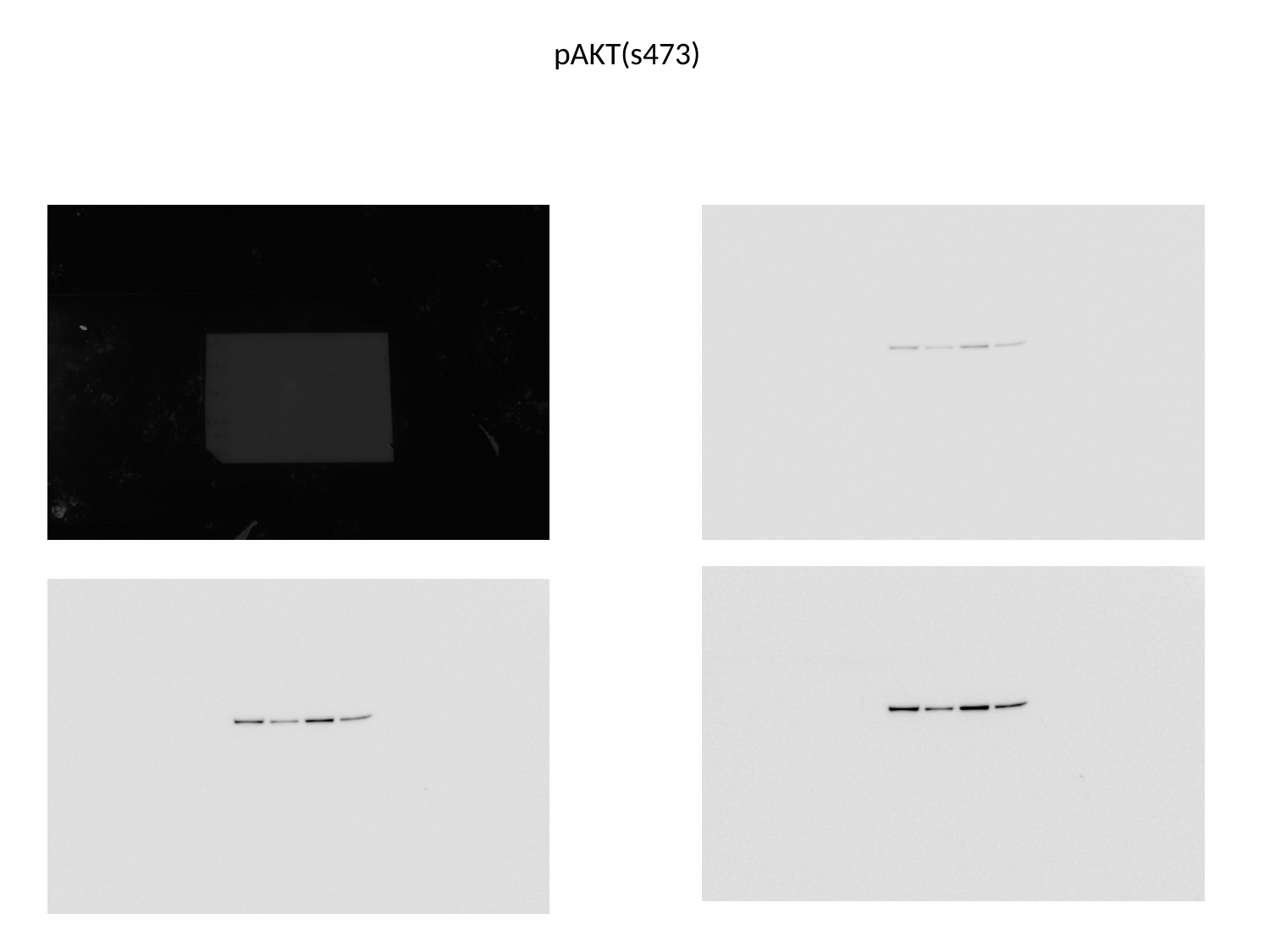

pAKT(s473)

## Slide 3
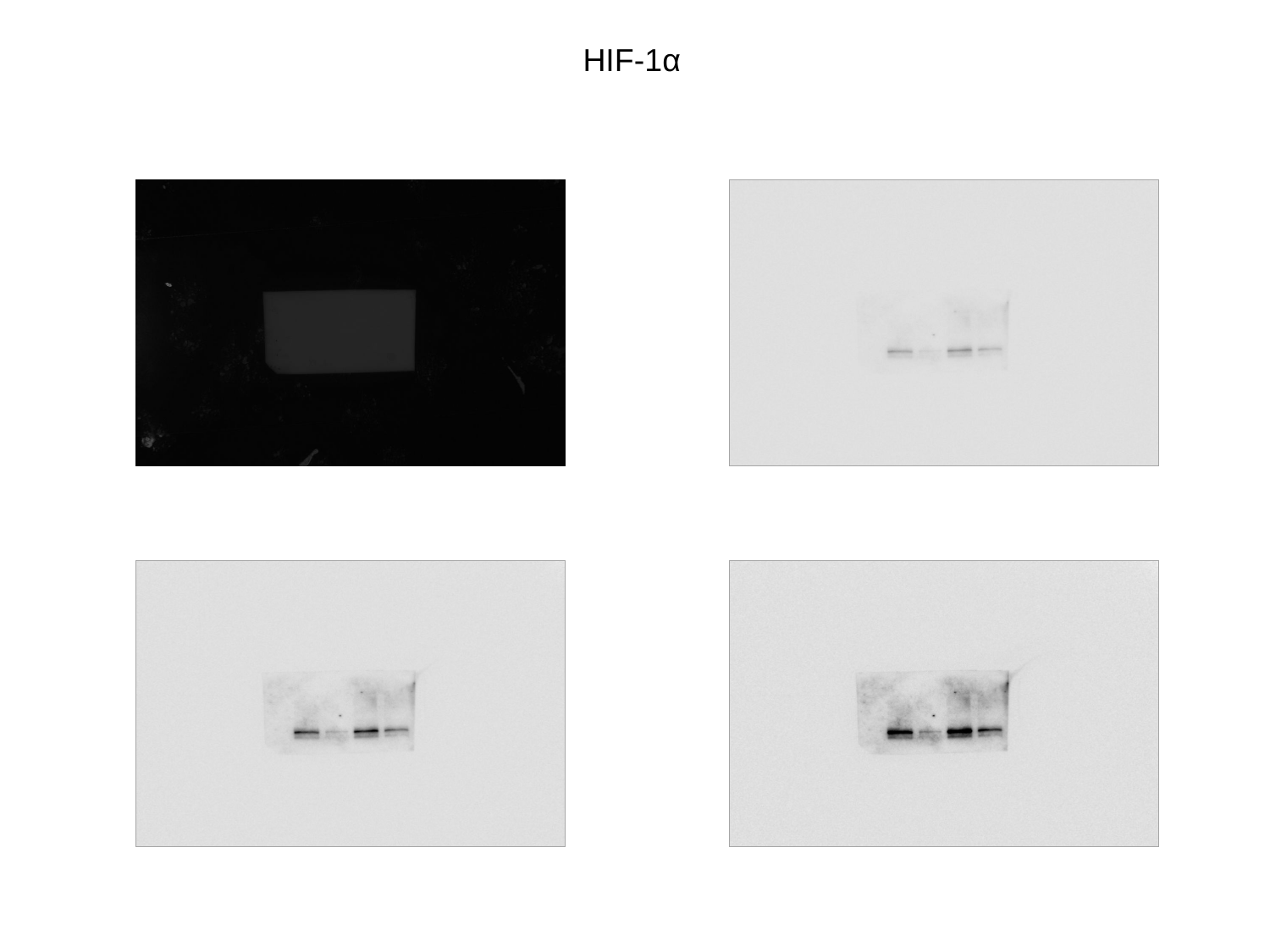

HIF-1α

## Slide 4
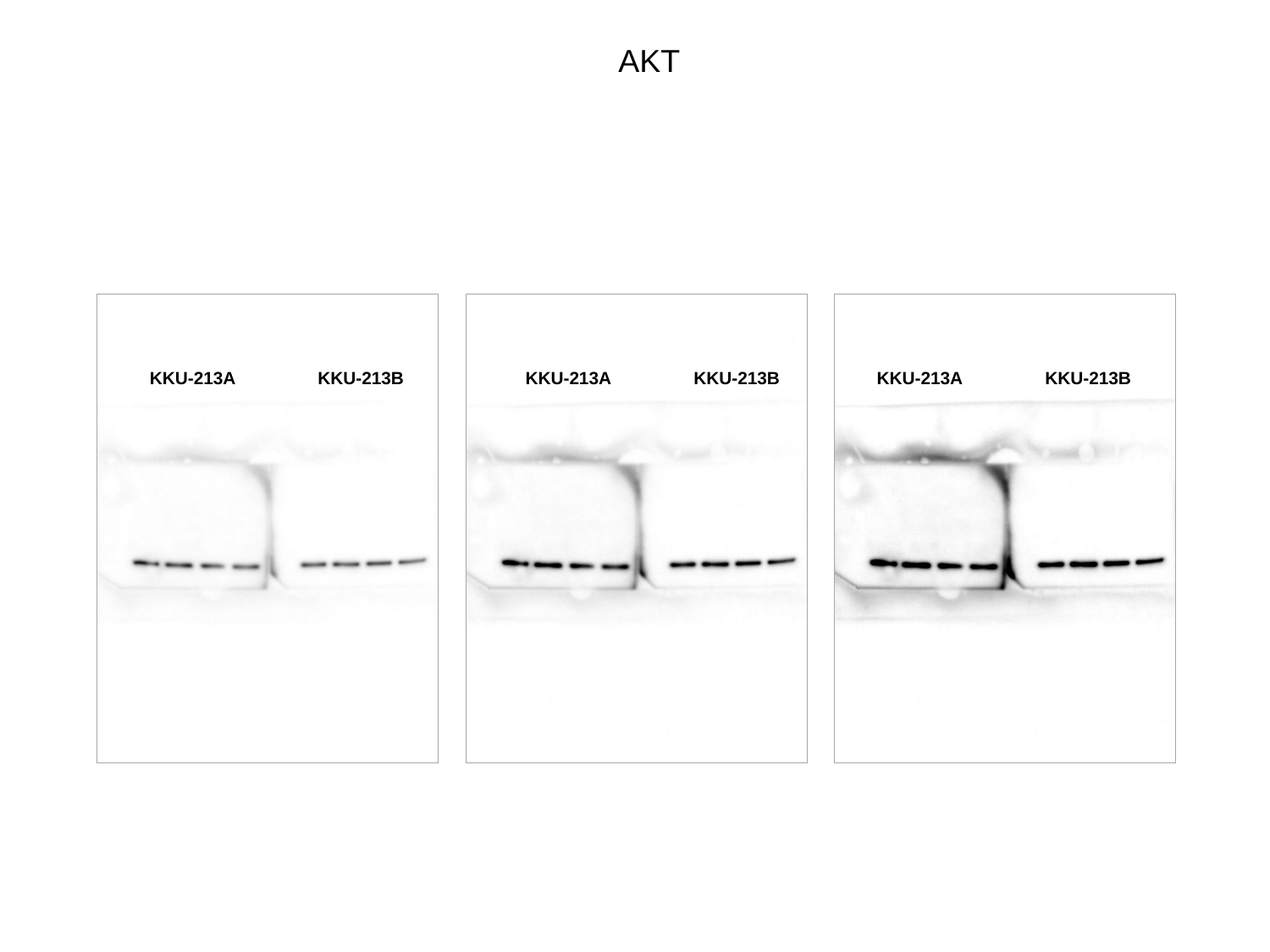

AKT
KKU-213A
KKU-213B
KKU-213A
KKU-213B
KKU-213A
KKU-213B

## Slide 5
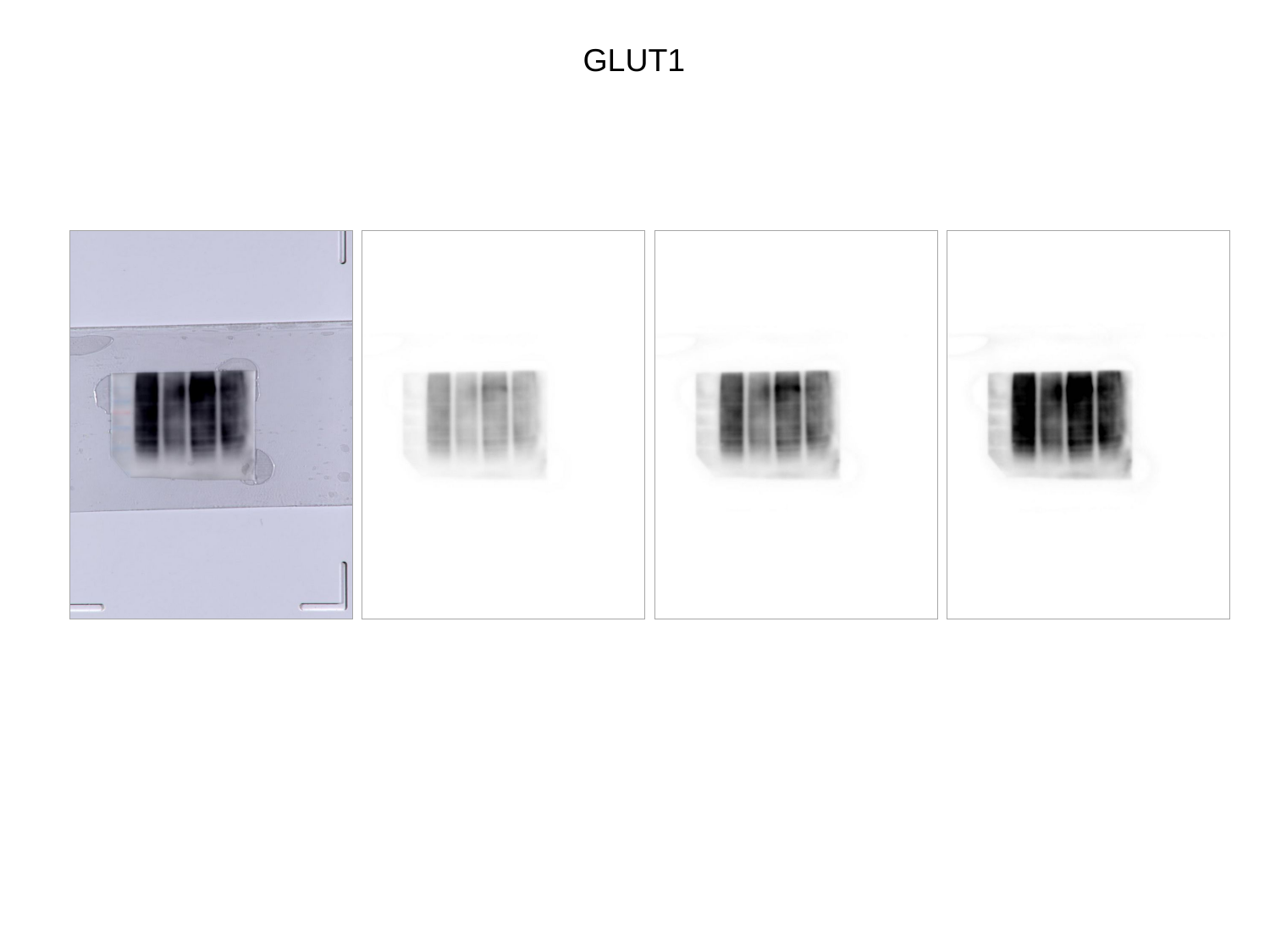

GLUT1

## Slide 6
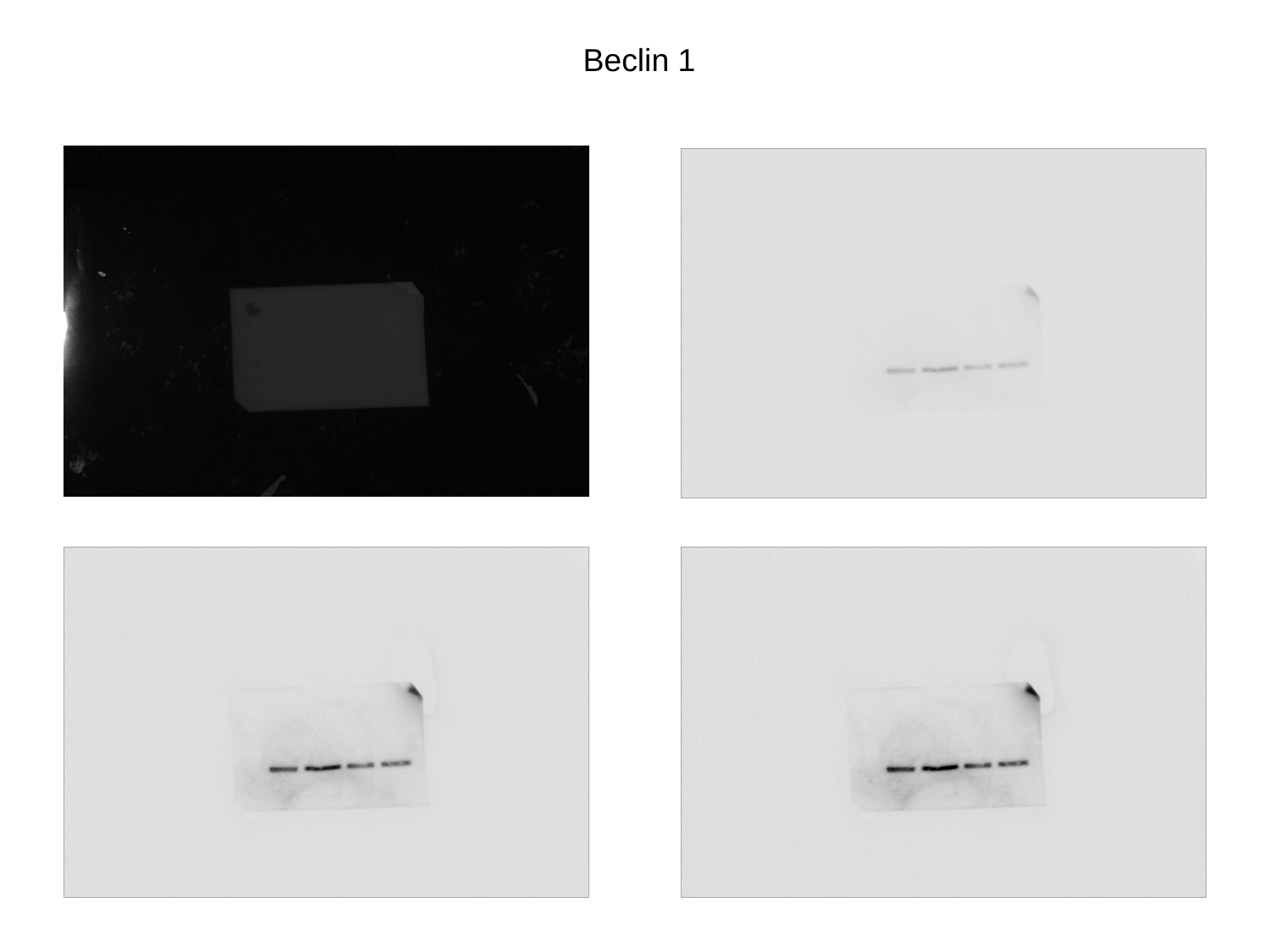

Beclin 1

## Slide 7
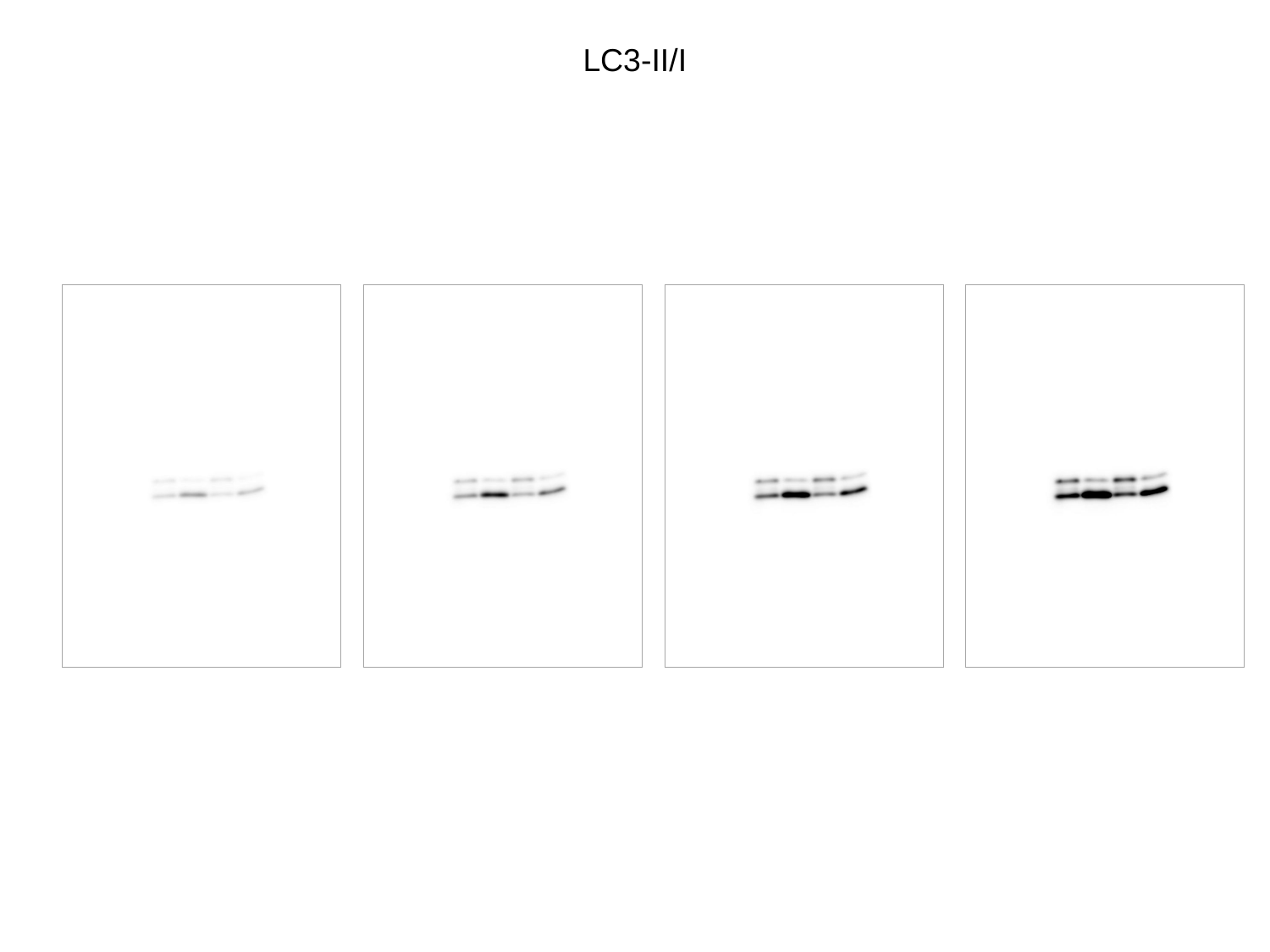

LC3-II/I

## Slide 8
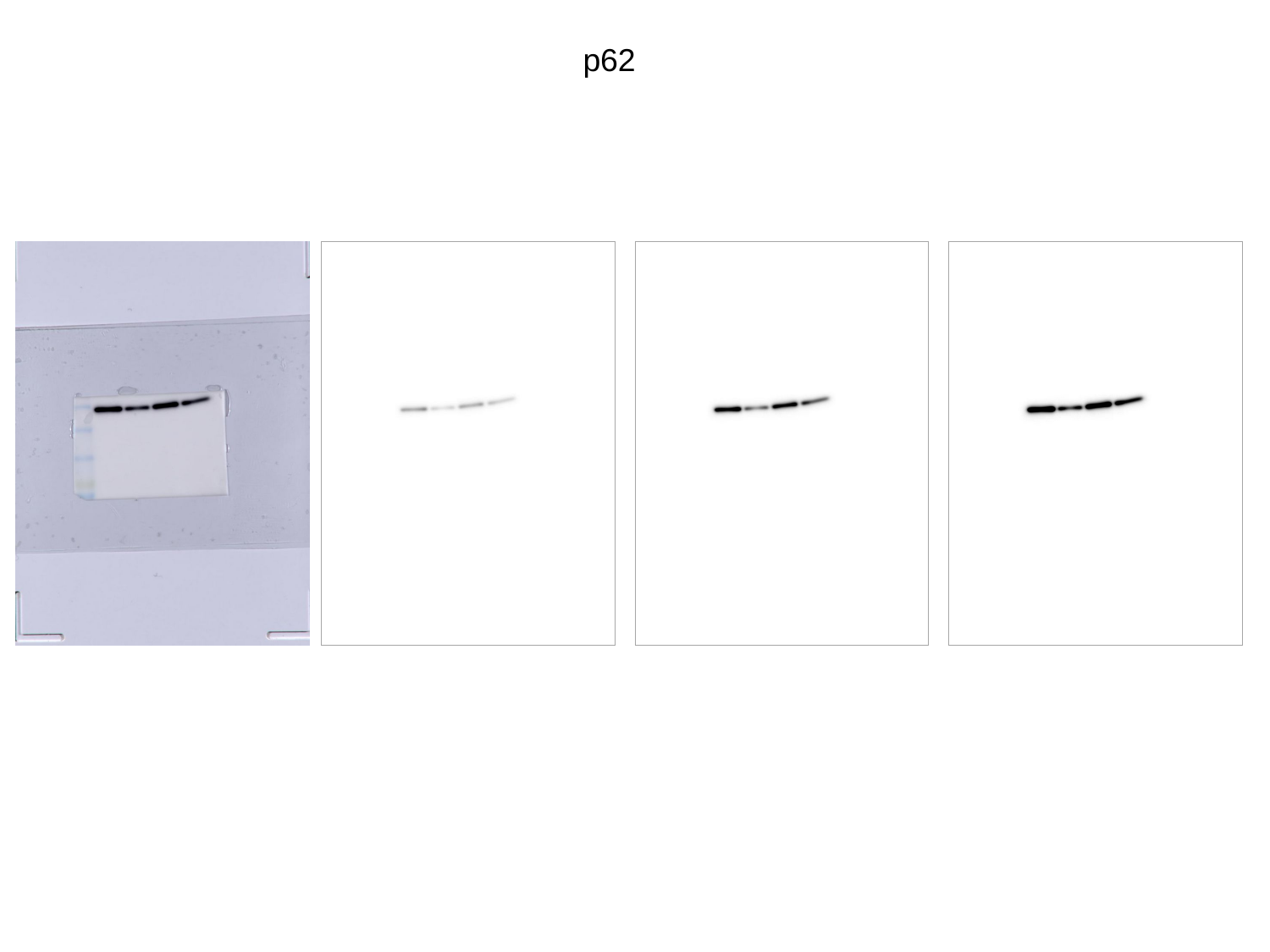

p62

## Slide 9
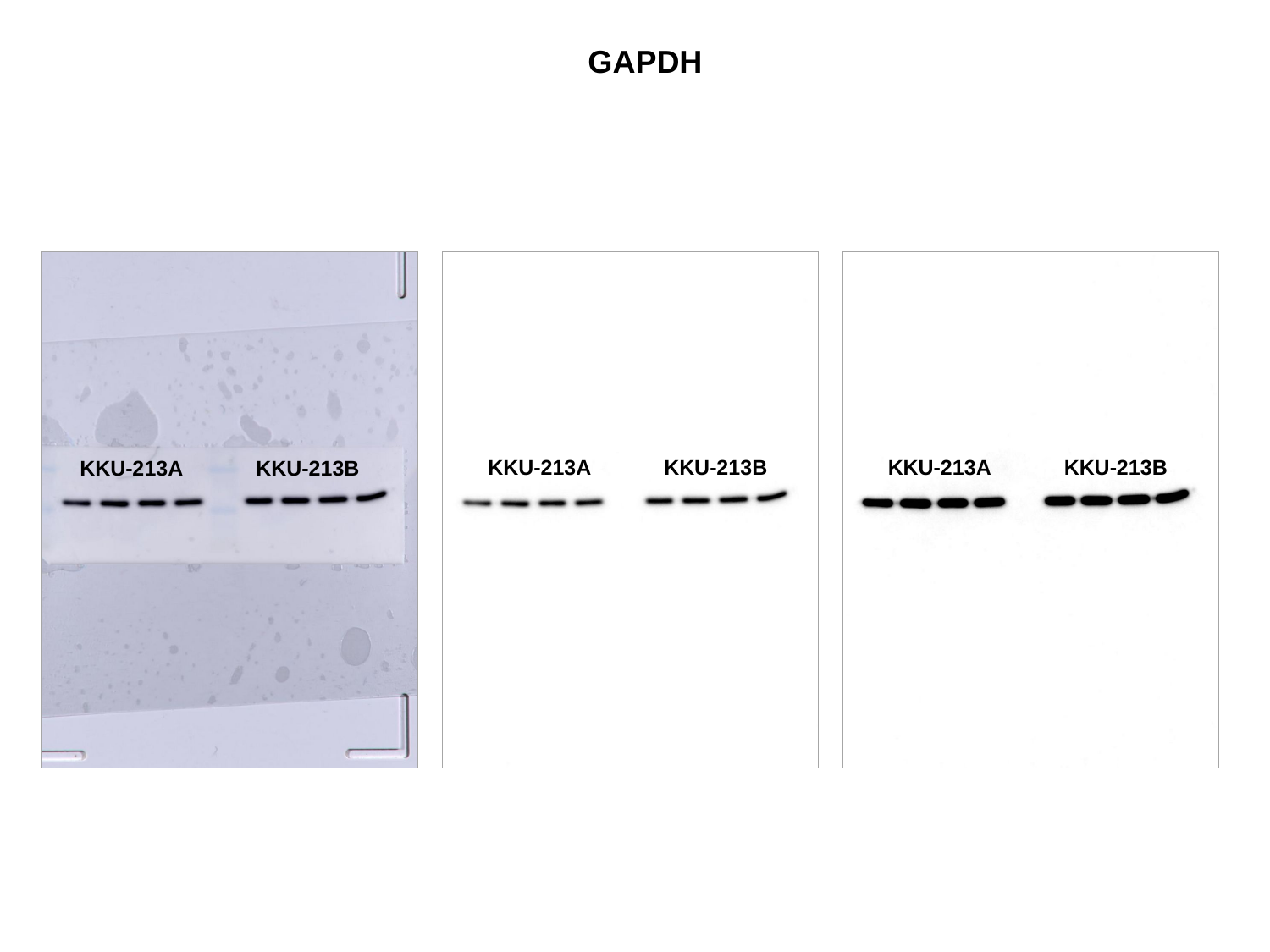

GAPDH
KKU-213A
KKU-213B
KKU-213A
KKU-213B
KKU-213A
KKU-213B

## Slide 10
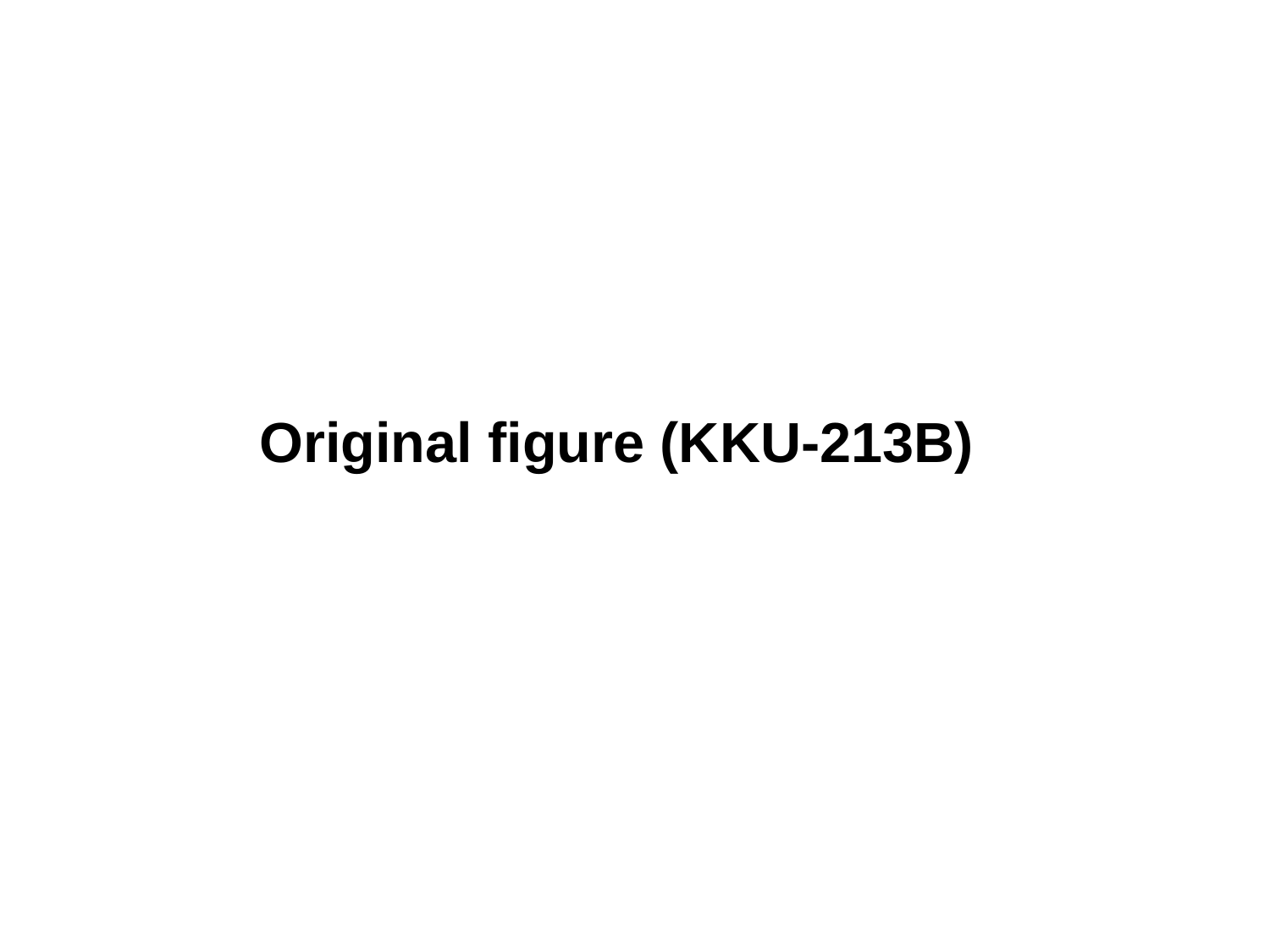

Original figure (KKU-213B)

## Slide 11
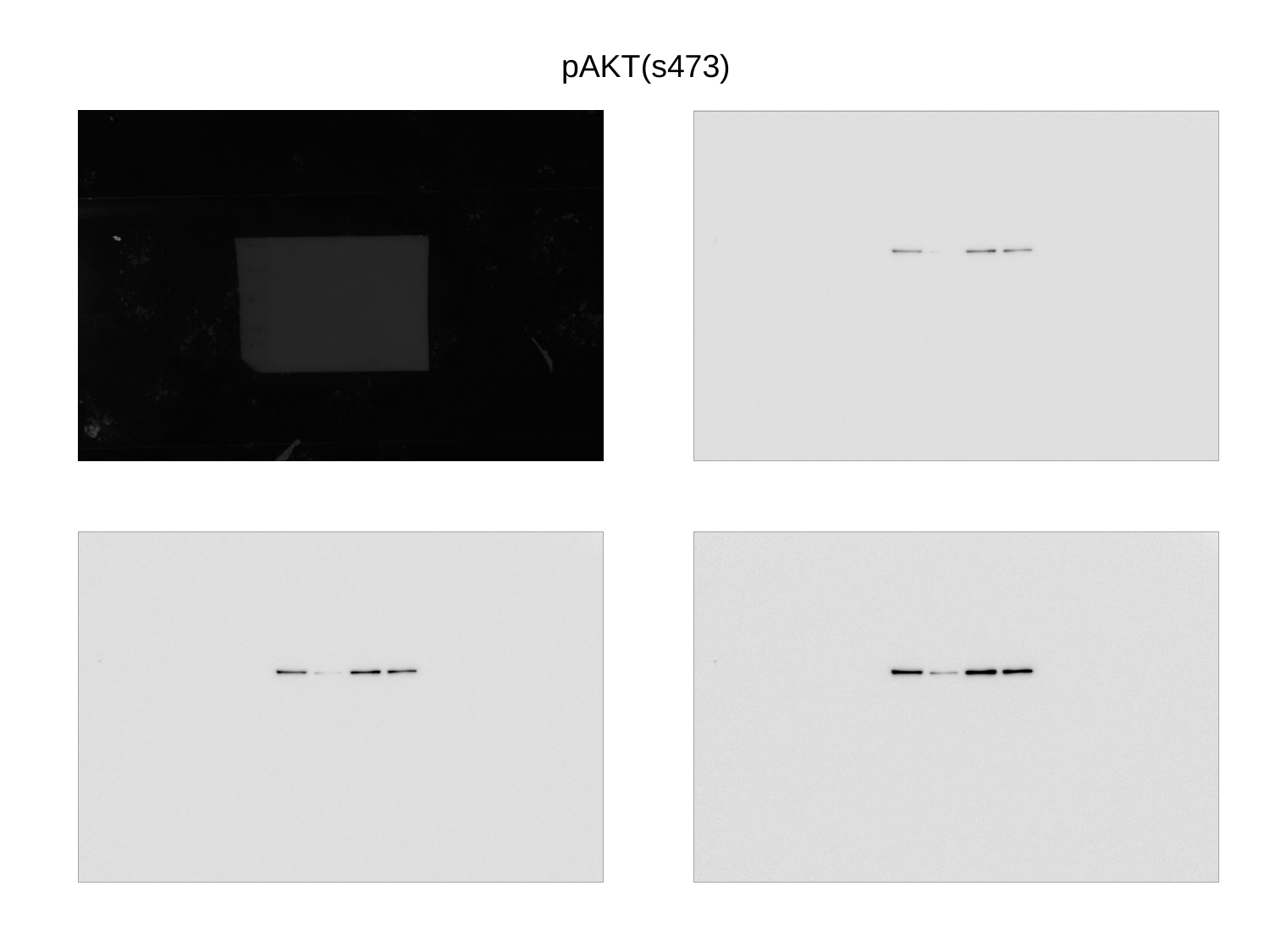

pAKT(s473)

## Slide 12
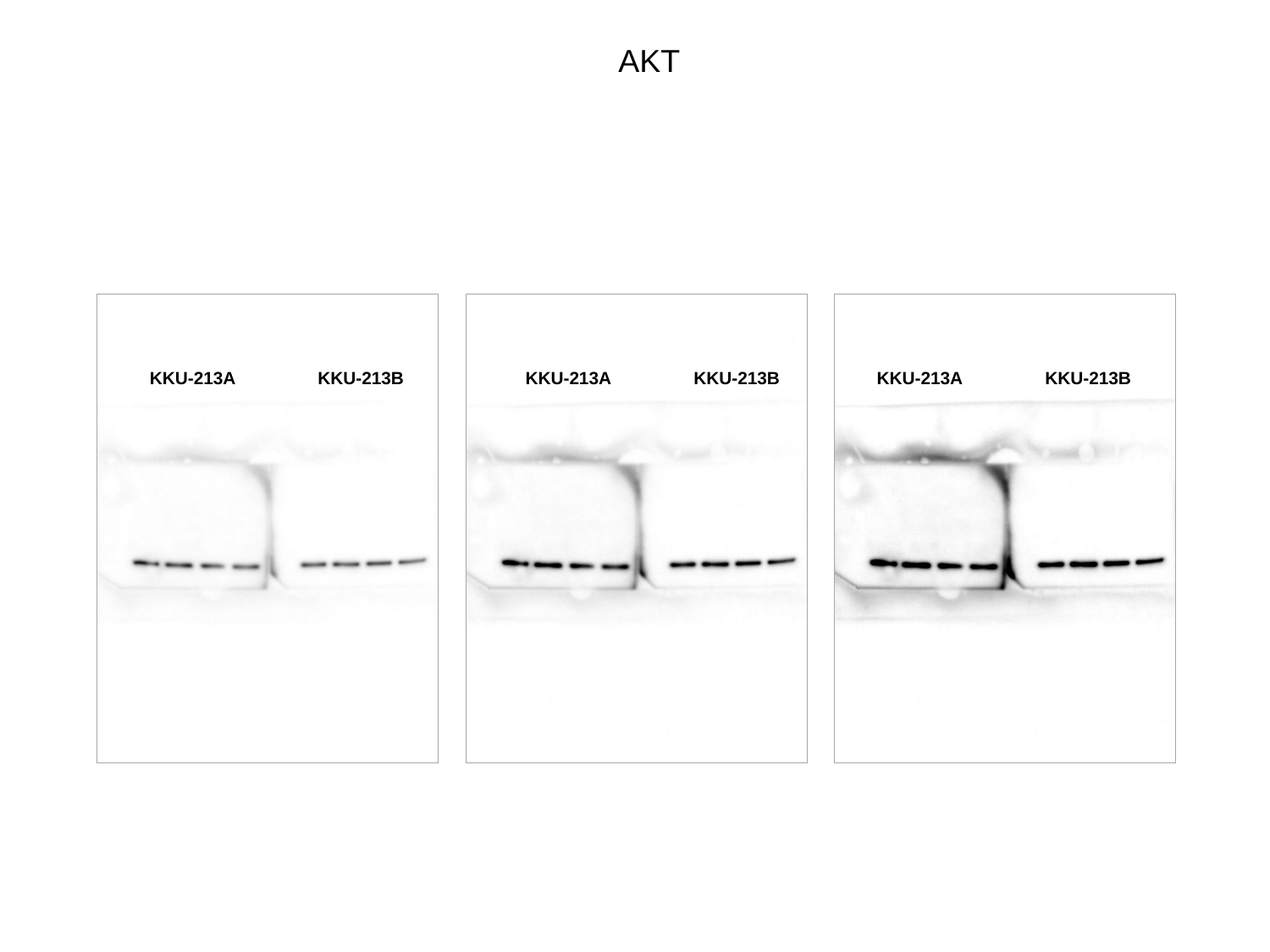

AKT
KKU-213A
KKU-213B
KKU-213A
KKU-213B
KKU-213A
KKU-213B

## Slide 13
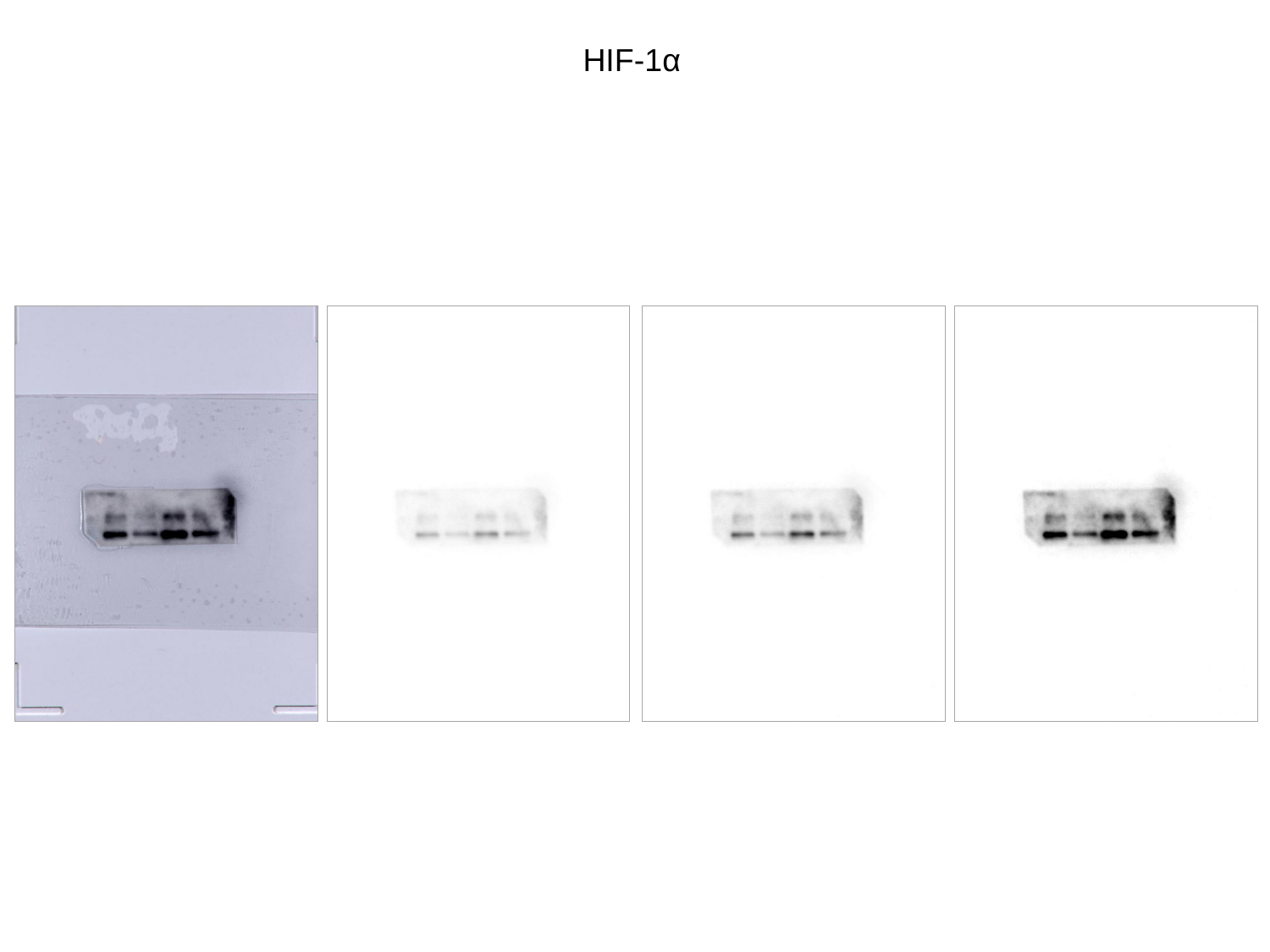

HIF-1α

## Slide 14
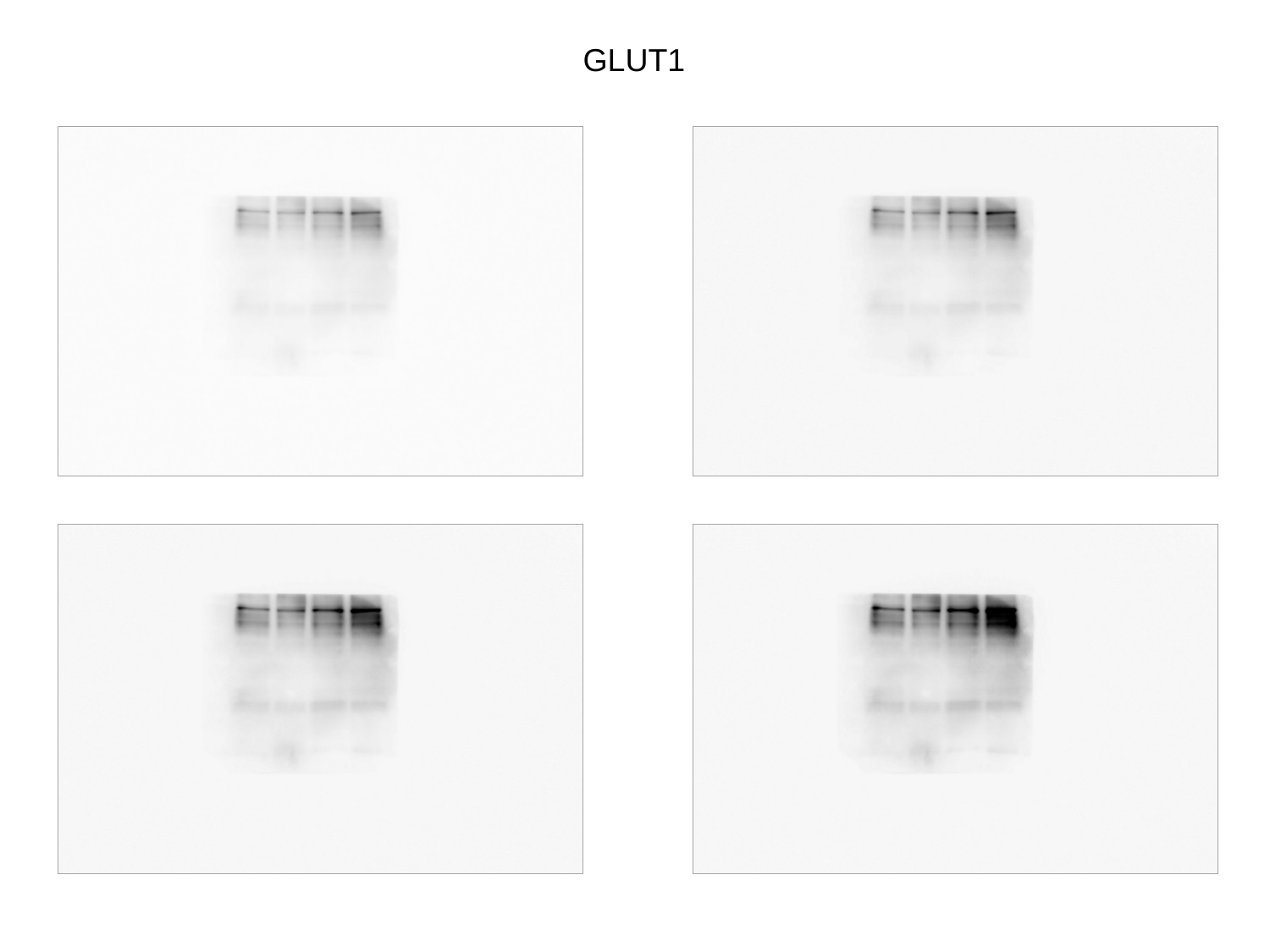

GLUT1

## Slide 15
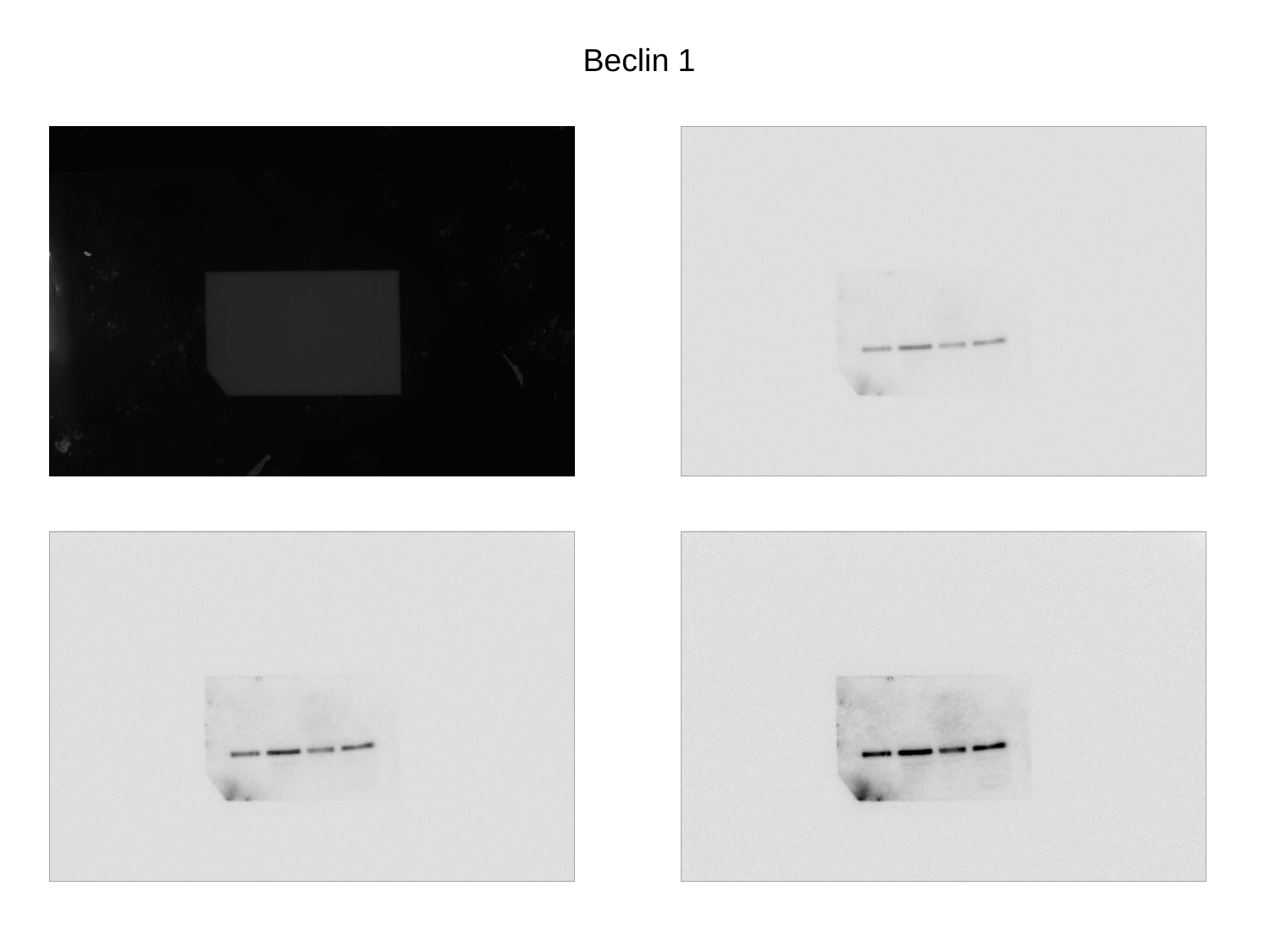

Beclin 1

## Slide 16
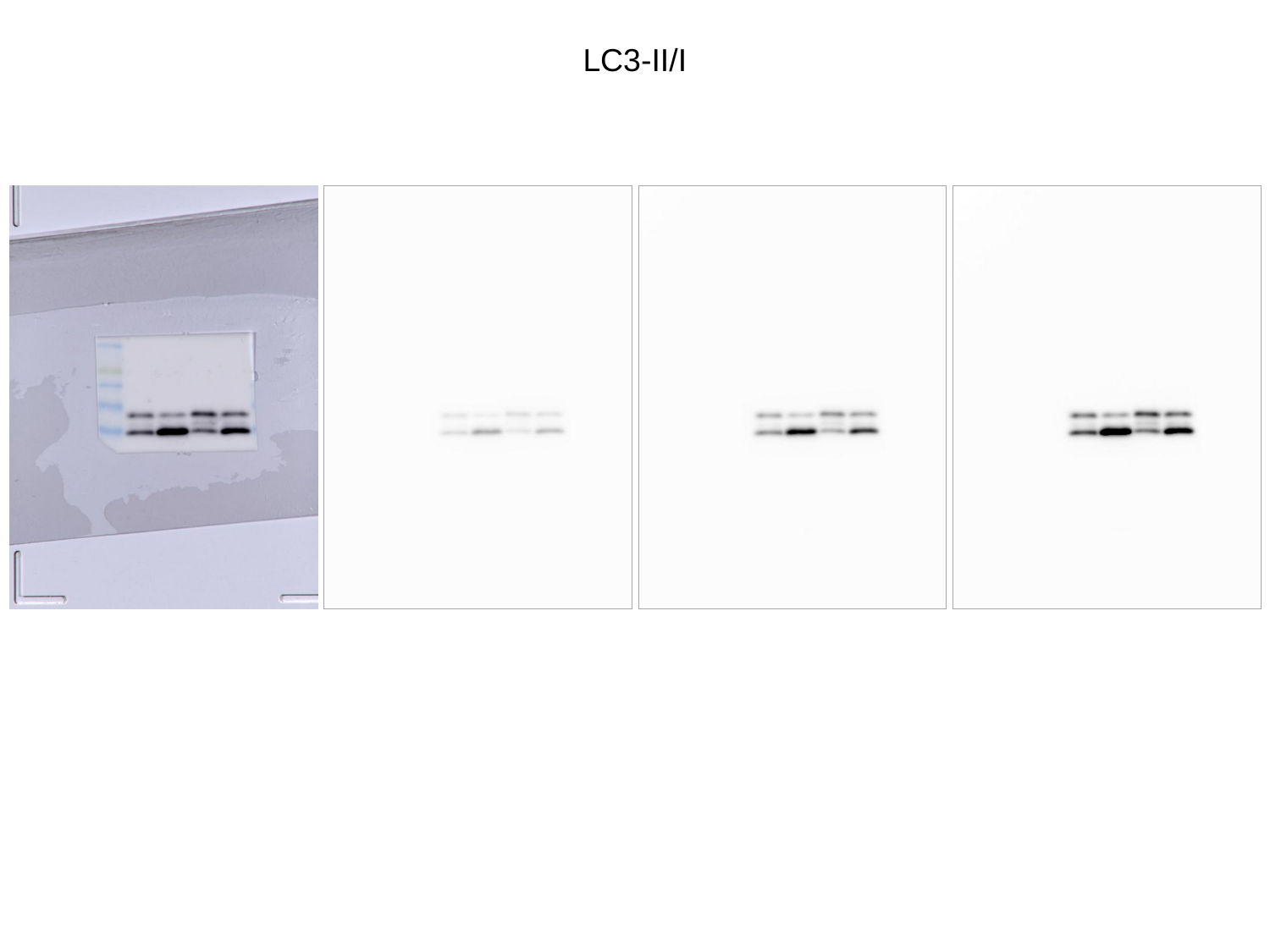

LC3-II/I

## Slide 17
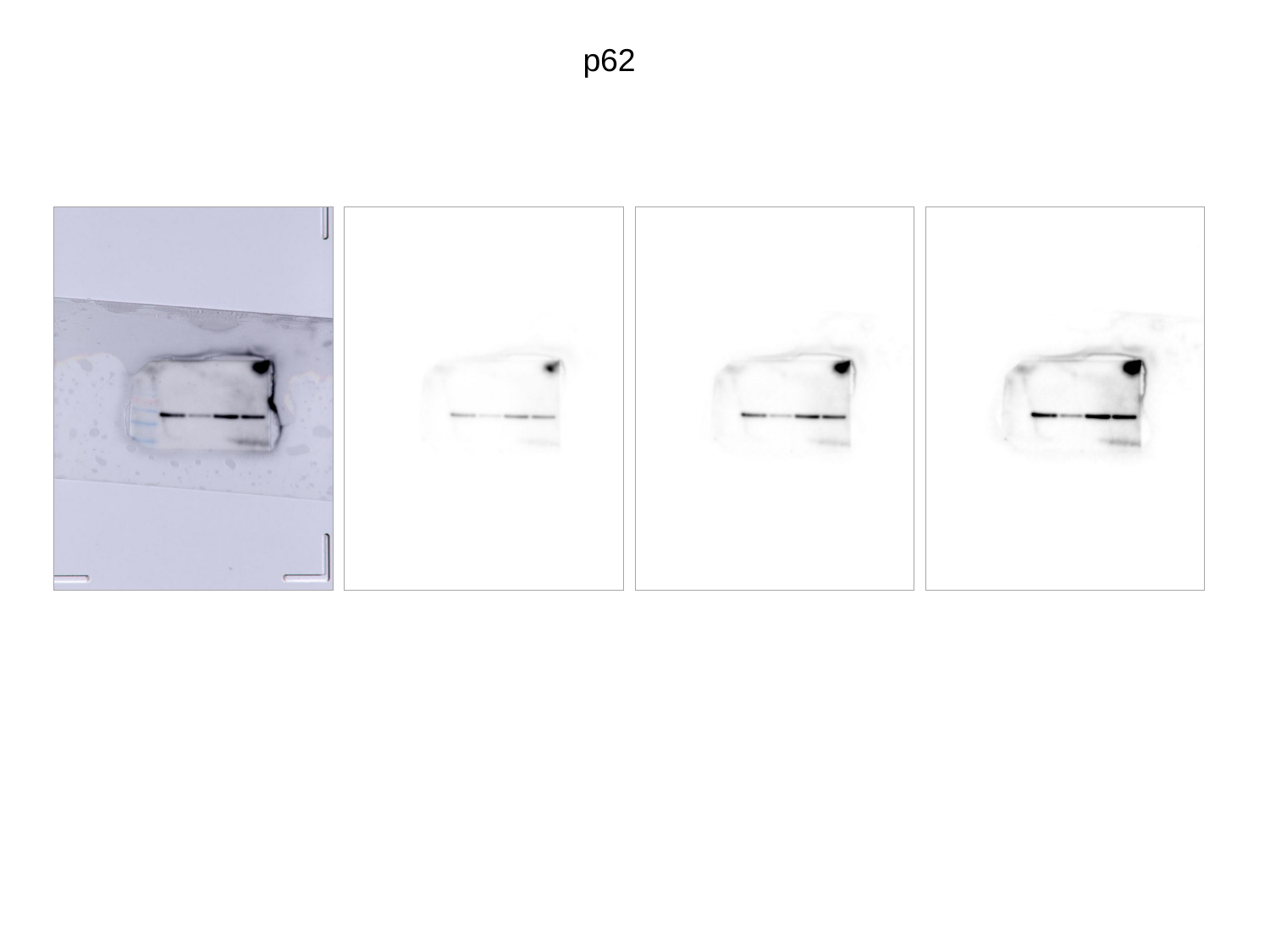

p62

## Slide 18
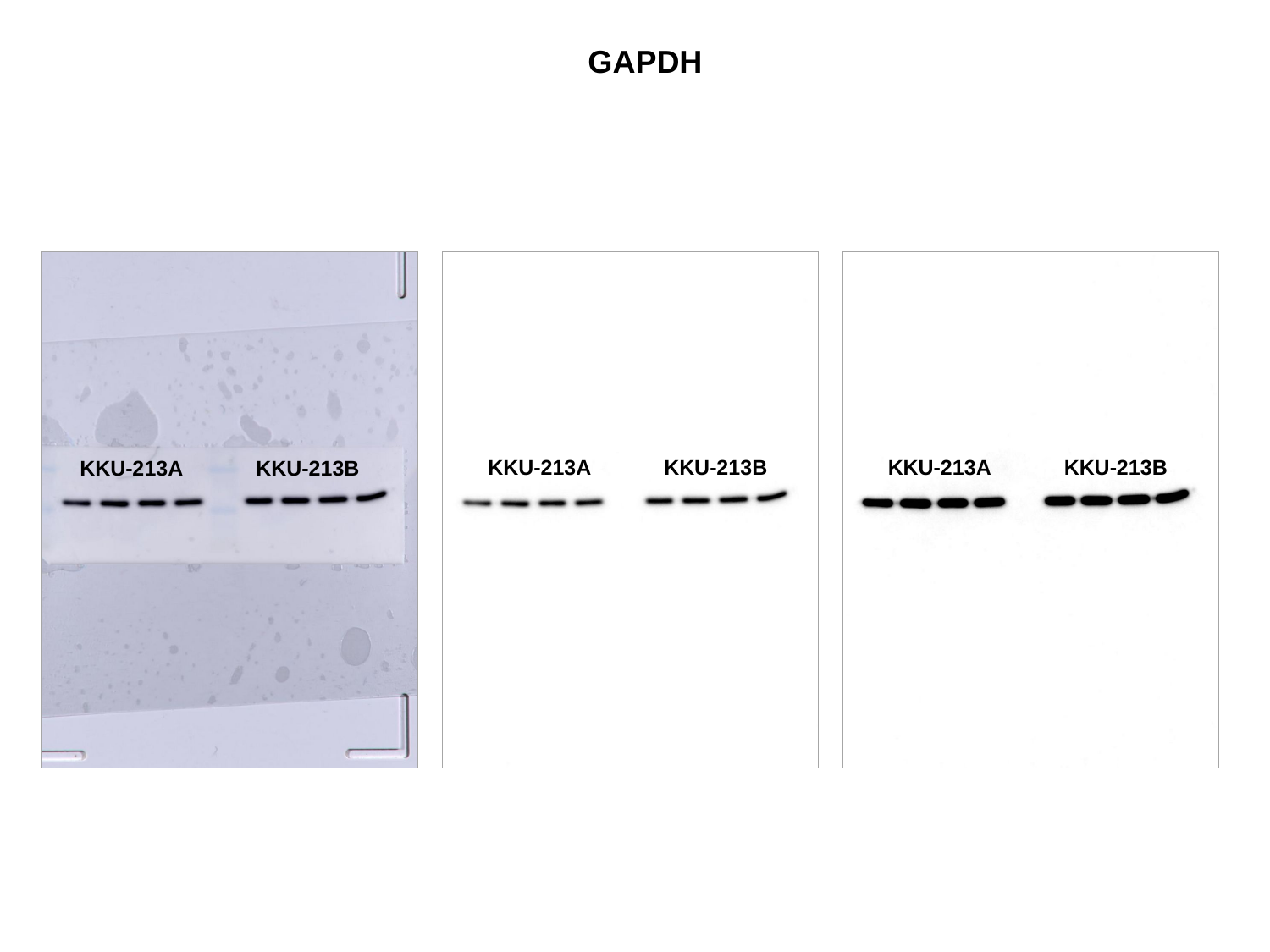

GAPDH
KKU-213A
KKU-213B
KKU-213A
KKU-213B
KKU-213A
KKU-213B
